# Polyhedral Cu_2_O to Cu pseudomorphic conversion for stereoselective alkyne semihydrogenation†
†Electronic supplementary information (ESI) available. See DOI: 10.1039/c7sc05232d


**DOI:** 10.1039/c7sc05232d

**Published:** 2018-01-31

**Authors:** Sourav Rej, Mahesh Madasu, Chih-Shan Tan, Chi-Fu Hsia, Michael H. Huang

**Affiliations:** a Department of Chemistry , National Tsing Hua University , Hsinchu 30013 , Taiwan . Email: hyhuang@mx.nthu.edu.tw

## Abstract

Cu_2_O cubes, octahedra, and rhombic dodecahedra can be pseudomorphically converted into Cu crystals of corresponding shapes through reduction by ammonia borane in ethanol at 50 °C or below within 3 min, demonstrating the feasibility of making challenging polyhedral metal particles from metal oxide crystals. Hydrogen gas is also produced from ammonia borane in the process. The obtained Cu crystals have a slightly nanoporous interior. Addition of diphenylacetylene in the formation of Cu rhombic dodecahedra leads to complete stereoselective production of sterically hindered (*Z*)-stilbene. Semihydrogenation of other alkynes also gives pure (*Z*)-alkenes. Cu cubes and octahedra also showed considerable (*Z*)-stilbene selectivity along with minor formation of (*E*)-stilbene and bibenzyl as compared to CuCl_2_ and commercial Cu_2_O particles. Mechanistic studies reveal that the low binding affinity of alkenes on the rhombic dodecahedra surfaces leads to high product selectivity. These Cu crystals act as a green and low-cost catalyst for the synthesis of high-purity (*Z*)-alkenes.

## Abstract 2

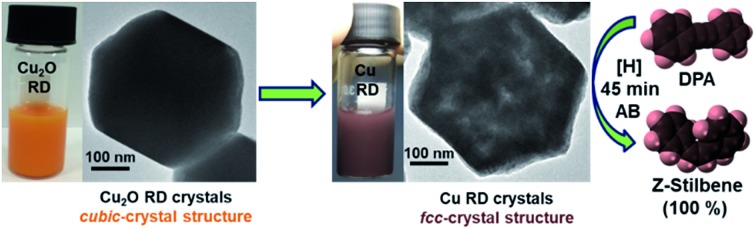
Cu_2_O nanocrystals can be pseudomorphically converted into Cu crystals through ammonia borane reduction, releasing hydrogen for stereoselective semihydrogenation of diphenylacetylene.

## Introduction

The catalytic activity of inorganic nanocrystals has been shown to greatly depend on the exposed surface facets.^1^–^5^ Considering the low-cost advantage of copper nanocrystals and their broad applications in various catalytic reactions, it is highly desirable to make copper nanocrystals with diverse shapes using benign reaction conditions.^6^ While cubic and octahedral copper nanocrystals have been prepared, copper rhombic dodecahedra have been unknown until recently.^7^–^11^ Cu_2_O crystals with these polyhedral shapes can be readily synthesized.^1^ Direct conversion of Cu_2_O polyhedra to copper particles of corresponding morphologies is highly useful, but such transformation has not been demonstrated until recently. Cu_2_O cubes, octahedra, and rhombic dodecahedra were synthesized using different reagents, including the addition of poly(vinylpyrrolidone) (PVP) to make octahedra.^11^ After a special heat treatment to remove PVP and oleic acid, the Cu_2_O crystals were heated to 548 K under a CO/N_2_ stream for 2 h to obtain Cu crystals for corresponding shapes. However, the Cu surfaces are rough and contain numerous pits. Sharp crystal faces are critically important for facet-dependent examinations. It is also interesting to learn that examples of using Cu-based nanomaterials for alkyne semihydrogenation reactions have not been available until recently.^6^,^12^ Stereoselective semihydrogenation of internal alkynes to thermodynamically unstable and sterically hindered pure (*Z*)-alkenes is a powerful tool in industrial organic synthesis and necessary to manufacture a wide variety of fine chemicals, drugs and bioactive natural products.^13^ Currently, the Lindlar catalyst has generally been used for (*Z*)-alkene synthesis by semihydrogenation of internal alkynes.^14^ This heterogeneous catalyst consists of costly palladium deposited on calcium carbonate and poisoned with lead acetate.^14^ A large amount of quinoline is also needed for higher product selectivity. The presence of toxic lead and excess quinoline are the biggest drawbacks of the Lindlar catalyst. To overcome this problem, many modified nanocatalysts have been developed recently, based on Pd,^15^–^21^ Ni,^22^,^23^ Au,^24^–^26^ and other materials.^27^,^28^ In spite of all these advances, sometimes a complex catalyst preparation and harsh reaction conditions make them very hard to be reproduced by others even for simple laboratory-scale applications. Hence, a very simple, easily reproducible, low cost, and green catalytic system is in high demand for synthesizing high-purity (*Z*)-alkenes.

In this work, we have shown that in the presence of ammonia borane (AB) and ethanol, well-defined cubic, octahedral, and rhombic dodecahedral Cu_2_O crystals with oxygen ions forming a body-centered cubic crystal lattice and copper ions filling half of the tetrahedral sites can be pseudomorphically converted into Cu crystals with face-centered cubic crystal structures maintaining the same particle shapes at room temperature. Pseudomorphic transformations from Cu_2_O polyhedra to Cu_2_S, CdS, and ZnS cages have been demonstrated.^29^,^30^ However, pseudomorphic conversion from a semiconductor to a metal has not been known until recently. The pseudomorphic transformation completes rapidly in a few minutes with *in situ* generated hydrogen from AB. Hydrogen evolution from AB decomposition in the presence of Au, Pd, and Cu nanocrystals has been reported.^31^,^32^ Cu rhombic dodecahedra showed 100% stereoselectivity towards the semihydrogenation of diphenylacetylene (DPA) to (*Z*)-stilbene in the presence of AB, although Cu cubes and octahedra also displayed considerable product selectivity. Comparison of the catalytic activity of Cu crystals to those of CuCl_2_, commercial Cu_2_O, and Lindlar catalyst has been made. The time-dependent formation of different products has also been studied. Mechanistic study reveals that the low binding affinity for alkenes is the main cause of the high stereoselectivity of RD Cu crystals.

## Results and discussion

### Pseudomorphic formation of Cu polyhedra

**Fig. 1:**
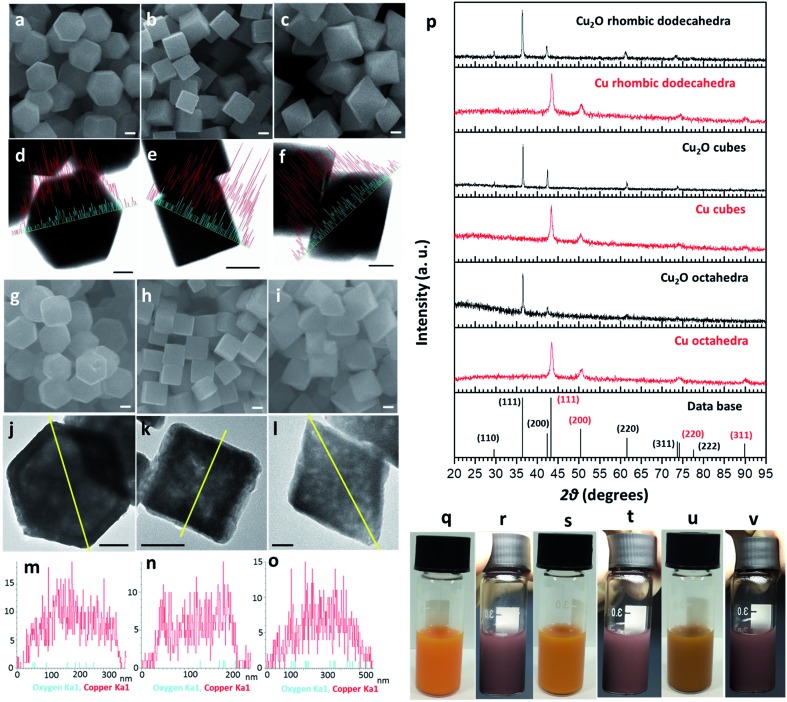
Pseudomorphic formation of Cu polyhedra from Cu_2_O crystals. (a–c) SEM and (d–f) HAABF-STEM images with EDS elemental line scans for Cu_2_O RD, cubic, and octahedral nanocrystals, respectively. The red and blue lines correspond to copper and oxygen, respectively. (g–i) SEM images, (j–l) HAABF-STEM images, and (m–o) EDS line scans across the yellow line for Cu RD, cubic, and octahedral nanocrystals, respectively. The red signals come from copper, while the blue signals are from oxygen. (p) XRD patterns of the Cu_2_O and Cu crystals. (q–v) Photographs of (q and r) RD, (s and t) cubic, and (u and v) octahedral Cu_2_O and Cu crystals. All scale bars are equal to 100 nm.

Cu_2_O crystals with rhombic dodecahedral, cubic, and octahedral shapes were synthesized at room temperature by reacting an aqueous solution of CuCl_2_ with NaOH and reducing Cu(OH)_2_ or Cu(OH)_4_^2–^ with hydroxylamine hydrochloride (Fig. 1a–c).^33^,^34^ High-angle annular bright-field scanning transmission electron microscopy (HAABF-STEM) images with energy-dispersive X-ray spectroscopy (EDS) line scans of rhombic dodecahedral, cubic, and octahedral Cu_2_O crystals show perfect particle geometries and that the crystals are composed of Cu and O atoms (Fig. 1d–f). To prepare Cu polyhedra, an ethanolic solution of the Cu_2_O crystals was treated with AB at room temperature for 5 to 9 minutes (Table S1, ESI†). Addition of AB as a reducing agent leads to remarkable pseudomorphic conversion of Cu_2_O crystals to Cu particles, showing the feasibility of metal polyhedra formation through direct one-pot pseudomorphic transformation of their corresponding metal oxides for the first time. When we used hydrazine as the reducing agent, many Cu rhombic dodecahedra showed etched faces. When sodium borohydride was used as the reducing agent, the resulting particles lost their original morphology (Fig. S1, ESI†). Scanning electron microscopy (SEM) images confirm pseudomorphic formation of Cu rhombic dodecahedra, cubes, and octahedra from Cu_2_O crystals of corresponding shapes (Fig. 1g–i). Size distribution histograms show that the obtained Cu crystals retain the sizes of the original Cu_2_O particles (Fig. S2, ESI†). The Cu rhombic dodecahedra, cubes, and octahedra have sizes of 262 ± 20, 183 ± 11, and 387 ± 45 nm, respectively. HAABF-STEM images reveal that the resultant Cu crystals contain some nanopores in the crystal interior (Fig. 1j–l). EDS line scans on these crystals give only intense and continuous signals for Cu. The oxygen signals are very weak and discontinuous and are considered only as the background noise (Fig. 1m–o), proving an essentially complete loss of oxygen ions in the crystal lattice after the reduction process. Powder XRD patterns (Fig. 1p) establish that the diffraction peaks for Cu_2_O cubes, octahedra, and rhombic dodecahedra (JCPDS #77-0199) have been replaced entirely with those of Cu with a fcc lattice (JCPDS #85-1326). The compositional change can be readily observed visually, and it is due to this obvious solution color change from orange Cu_2_O crystals to reddish brown Cu particles that further structural analysis of the resulting crystals was performed (Fig. 1q–v). UV-visible spectra of the three Cu_2_O particle shapes and their corresponding Cu products are provided in Fig. S3, ESI.† Due to the large particle sizes, light scattering effects dominate the absorption feature in the near-infrared region for Cu_2_O crystals. The synthesized Cu crystals give a distinctive surface plasmon resonance peak in the range of 625 to 650 nm.^35^ Light scattering effects in the near-infrared region are also obvious because of their large particle sizes. X-ray photoelectron spectroscopy (XPS) analysis on Cu_2_O and Cu rhombic dodecahedral crystals shows only slight shifts in the Cu 2p peaks from 932.1 and 951.9 eV in Cu_2_O crystals to 932.3 and 952.1 eV in Cu crystals with an absence of satellite peaks (Fig. S4 and S5, ESI†).^9^,^36^ However, more distinct differences from the Cu LMM region can be used to differentiate Cu_2_O crystals from Cu crystals.^37^

**Fig. 2:**
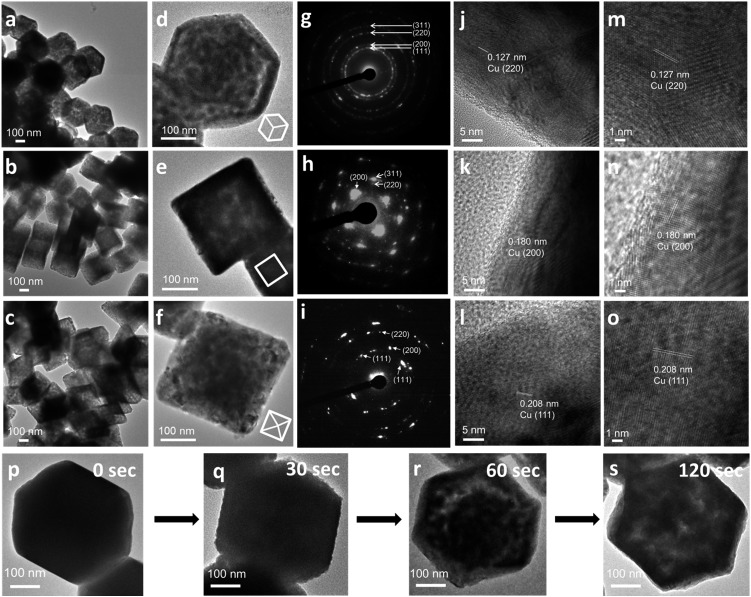
TEM characterization of Cu crystals. (a–c) TEM images, (d–f) single-particle TEM images with their orientations shown in white models, (g–i) corresponding SAED patterns, (j–l) HR-TEM images, and (m–o) magnified HR-TEM images of the near edges of Cu RD, cubic, and octahedral crystals, respectively. (p–s) Time-dependent TEM images showing how a Cu_2_O rhombic dodecahedron transforms into a Cu rhombic dodecahedron with intermediate particles collected at the specific time points indicated.

Large-area and single-particle transmission electron microscopy (TEM) images of Cu rhombic dodecahedra, cubes, and octahedra reveal more details of the internal structures of the Cu crystals (Fig. 2a–f). The particles appear slightly porous. However, compared to the transformation from polyhedral Cu_2_O crystals to Cu_2_S hollow cages upon Na_2_S addition, the Cu crystals retain most of their solid interior.^28^,^29^ Selected-area electron diffraction (SAED) patterns (Fig. 2g–i) taken on a single Cu rhombic dodecahedron give a ring pattern indicating the polycrystalline nature of the particle. However, the Cu cube and octahedron are largely single-crystalline. An interesting 2-fold symmetrical SAED pattern was recorded for the Cu octahedron. The acidic solution condition in the preparation of Cu_2_O rhombic dodecahedra naturally makes some particles slightly etched. It is unclear if this structural feature leads to a more local rather than uniform rate of the reduction process giving polycrystalline Cu rhombic dodecahedra. The large-area and magnified HR-TEM images of a Cu rhombic dodecahedron, cube, and octahedron reveal lattice fringes with interplanar spacings of 1.27, 1.80, and 2.04 Å corresponding to the (220), (200), and (111) lattice planes of fcc Cu (Fig. 2j–o). When lattice fringes close to the edge of the rhombic dodecahedron are examined (Fig. S6, ESI†), the planes perpendicular to the crystal edge yield a *d*-spacing consistent with the (110) planes of Cu, suggesting (110) planes are exposed in the Cu rhombic dodecahedra. (111) planes have also been found to align perpendicular to the edge of an octahedron (Fig. S7, ESI†). Fig. 2 also shows time-dependent TEM images illustrating how Cu_2_O rhombic dodecahedra are converted into Cu rhombic dodecahedra. The particle surfaces become slightly rough 30 s after the addition of AB. The reduction process occurs so rapidly that after 60 s a great extent of the particle interior appears porous, but the central portion seems less affected. The reduction process is mostly completed within 2 min after AB addition to give a more uniformly nanoporous appearance. Fig. S8, ESI† provides a schematic diagram for the mechanism of this pseudomorphic transformation. In ethanol, the BH_3_ unit from AB undergoes ethanolysis and releases hydride (H^–^) ions, which synchronously reduces Cu^+^–NH_3_ units to Cu metal whilst liberating NH_3_ and hydrogen gas molecules. Inductively coupled plasma mass spectrometry (ICP-MS) analysis gave a Cu concentration of ∼0.039 mg L^–1^ from the top solution after the formation of Cu rhombic dodecahedra. This low concentration of Cu in the solution indicates complete reduction of Cu^+^ ions in the crystals to Cu^0^ atoms.

### Stereoselective semihydrogenation of alkynes

**Fig. 3:**
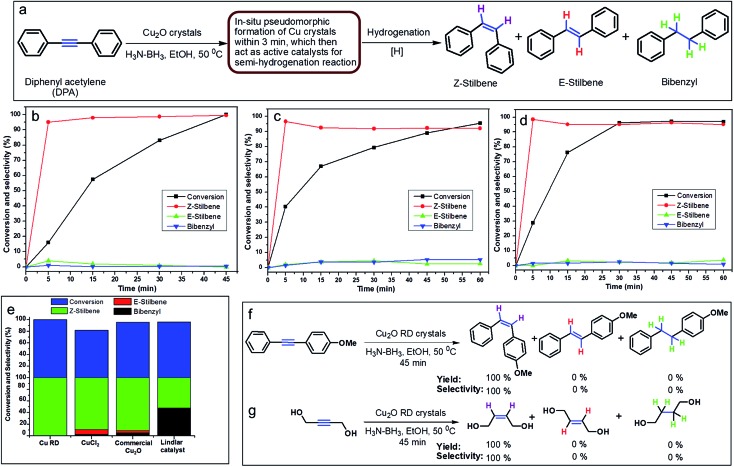
Results of the catalytic semihydrogenation reaction. (a) A schematic diagram for the stereoselective semihydrogenation of DPA by *in situ* formed Cu crystals. (b–d) Time-dependent plots for the conversion of DPA for Cu (b) rhombic dodecahedra, (c) cubes, and (d) octahedra. (e) Catalytic comparison of the Cu rhombic dodecahedra with CuCl_2_, commercial Cu_2_O, and Lindlar catalyst. (f and g) General applicability of the Cu rhombic dodecahedra toward different substituted alkynes.

**Fig. 4:**
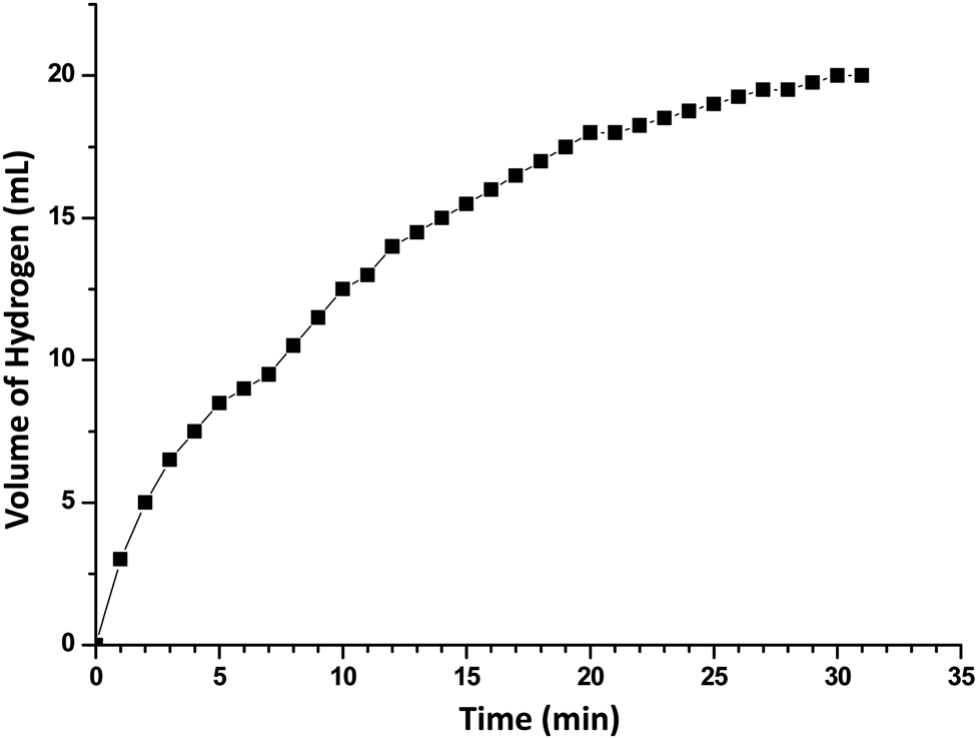
A representative plot for the volume of H_2_ generated from ammonia borane ethanolysis as a function of reaction time in the presence of Cu_2_O rhombic dodecahedra. Reaction conditions: catalyst = 4 mg, amount of ammonia borane used = 0.0123 g, temperature = 30 °C. The volume of hydrogen evolved is 20 mL in 31 min.

In order to develop an easily reproducible, one-pot, low-cost green catalytic system for stereoselective semihydrogenation of DPA to (*Z*)-stilbene, Cu_2_O crystals exposing different facets have been employed as the catalysts. DPA and an anhydrous ethanolic solution of AB were added simultaneously to an Ace pressure tube (see Fig. S9, ESI†). Within 3 min, the *in situ* pseudomorphically formed Cu crystals then act as the active catalyst for this semihydrogenation reaction without isolation (Fig. 3a). In this whole process, ammonia borane, which is a high-density hydrogen storage material,^38^ acts as a reducing agent for Cu crystal formation and then produces the quantitative hydrogen gas required for the catalytic reaction (Fig. 4). It should be mentioned that hydrogen is present in the form of hydrogen atoms on the copper surface from the dissociation of ammonia borane, which initiate the semihydrogenation reaction. So there is no need to dissociate the H_2_ molecule, which generally requires a higher activation energy on the Cu surface. This is the advantage of our reaction by which one can easily overcome the higher activation energy for H_2_ dissociation on the Cu surface. Conversion of DPA and the concentrations of the three different products at different time intervals were studied for the Cu rhombic dodecahedra (Fig. 3b), cubes (Fig. 3c), and octahedra (Fig. 3d). In all these studies, the proton nuclear magnetic resonance (^1^H-NMR) spectrum of the crude reaction product mixture was taken without performing any column chromatography to determine the yields of the different products at different time points. As evidenced by time-dependent ^1^H-NMR spectra (Fig. S10, ESI†), Cu rhombic dodecahedra showed the highest conversion (100%) of DPA in 45 min at 50 °C and the highest product selectivity yielding 99.5% of (*Z*)-stilbene using 0.4 mmol of ammonia borane. Lowering the amount of ammonia borane added gave lower product conversion percentages (see Table S2†). After the reaction, the Cu rhombic dodecahedra kept their well-defined shape and nanoporous interior structure (Fig. S11, ESI†). XRD patterns gave only Cu reflection peaks (Fig. S12, ESI†). The XPS spectrum taken after 3 min of the semihydrogenation reaction shows the characteristic Cu LMM peak feature (Fig. S13, ESI†). ICP-MS analysis gave a Cu concentration of ∼0.139 mg L^–1^ from the clear reaction mixture after 45 min of reaction (as shown in Fig. S9, ESI†). This low concentration of Cu in the solution further confirms the high stability of Cu RD crystals and the heterogeneous nature of the catalytic reaction without any contribution from dissolved Cu ions. In addition, a hot filtration test was also performed. After 15 min of reaction, we filtered the reaction solution and removed all the catalyst carefully. The filtered reaction mixture was kept at 50 °C for 30 min under constant stirring, but no increase in product formation was observed, which clearly confirmed the heterogeneous nature of the reaction. We also performed a blank test where only ammonia borane and DPA were used to carry out the semihydrogenation reaction at 50 °C for 45 min without using any Cu_2_O catalyst, but no conversion of DPA was observed.

To test the recyclability of the catalyst, we have carried out one more catalytic cycle under the standard conditions using the same Cu rhombic dodecahedral catalyst isolated from the first catalytic cycle (see Fig. S14, ESI†). The DPA conversion was 93.5%, whereas the selectivity for (*Z*)-stilbene, (*E*)-stilbene, and bibenzyl is 93.4%, 5.6% and 1%, respectively. The SEM image of the particles taken after the second catalytic cycle shows that the Cu RD crystals maintain their shape. When the reaction was carried out at room temperature (25 °C), Cu rhombic dodecahedra took just 90 min to complete the reaction with 97.2% conversion of DPA and 98.2% selectivity for (*Z*)-stilbene (Fig. S15, ESI†). For Cu cubes at 50 °C, conversion of DPA dropped to 95.6% after 60 min of reaction with a relatively low (*Z*)-stilbene selectivity of 92.1% (Fig. 3c and S16, ESI†). Cu octahedra showed 97% DPA conversion and 95.1% selectivity for (*Z*)-stilbene after 60 min of reaction (Fig. 3d and S17, ESI†). Both Cu cubes and octahedra also appeared to be good catalysts considering the simple and mild reaction conditions.

We also evaluated the catalytic activity and product selectivity of CuCl_2_, commercial Cu_2_O and Lindlar catalyst for the same reaction (Fig. 3e). CuCl_2_ (see Fig. S18, ESI†) and commercial Cu_2_O (Fig. S19, ESI†) showed a conversion of DPA of 81.5% and 95.5% with comparatively lower selectivity for (*Z*)-stilbene of 89.2% and 90.9%, respectively. Commercial Lindlar catalyst (Fig. S20, ESI†) in the presence of hydrogen gas and quinoline displayed 52% selectivity towards (*Z*)-stilbene with 96% conversion of DPA with 48% undesired bibenzyl formation (Fig. S21, ESI†). When AB was used to provide hydrogen gas in the absence of quinoline, Lindlar catalyst produced only bibenzyl as the exclusive product after 15 min of reaction and has lost its selectivity for (*Z*)-stilbene (Fig. S22, ESI†).

Cu_2_O conversion to Cu crystals has been tested using different reducing agents such as hydrazine (Fig. S23, ESI†) and sodium borohydride (Fig. S24, ESI†) with the Cu_2_O RD catalyst. The conversion of DPA for hydrazine and sodium borohydride dropped to only 7.8% and 36.5% with 50.2% and 90.1% selectivity for (*Z*)-stilbene, respectively. The results show the importance of AB in this reaction. Table 1 summarizes all the catalysis results. Cu rhombic dodecahedra in conjunction with AB are the best catalytic system for the semihydrogenation of internal alkynes with an unsurpassed conversion and stereoselectivity. To further demonstrate the general applicability of the Cu rhombic dodecahedra, stereoselective semihydrogenation of 1-methoxy-4-(phenylethynyl)benzene (Fig. 3f and S25, ESI†) and 1,4-butynediol (Fig. 3g and S26, ESI†), an aliphatic molecule, has been carried out. In both cases, the DPA conversion was 100% with remarkably 100% selectivity for (*Z*)-alkenes in just 45 min.

**Table 1:** Comparison of catalytic activity and product selectivity over different catalysts used in the semihydrogenation of DPA. Amounts of different products are calculated directly from the ^1^H-NMR spectra of the crude reaction product without performing any column chromatography. Turnover frequency (TOF) values are also provided

Entry	Catalyst	Reducing agent	Time (min)	Temperature	Conversion of DPA	*Z*-Stilbene	*E*-Stilbene	Bibenzyl	TOF (min^–1^)
1	Cu_2_O RD crystals	H_3_N–BH_3_	45	50 °C	100%	99.5%	0%	0.5%	0.039
2	Cu_2_O cubic crystals	H_3_N–BH_3_	60	50 °C	95.6%	92.1%	2.7%	5.2%	0.028
3	Cu_2_O octahedral crystals	H_3_N–BH_3_	60	50 °C	97%	95.1%	3.8%	1.1%	0.028
4	Cu_2_O RD crystals	H_3_N–BH_3_	90	25 °C	97.2%	98.2%	1.0%	0.8%	0.019
5	CuCl_2_	H_3_N–BH_3_	60	50 °C	81.5%	89.2%	8.4%	2.4%	0.024
6	Commercial Cu_2_O	H_3_N–BH_3_	45	50 °C	95.5%	90.9%	4.1%	5%	0.037
7	Lindlar catalyst + quinoline (100 μL)	H_2_	30	50 °C	96%	52%	0%	48%	0.057
8	Lindlar catalyst	H_3_N–BH_3_	15	50 °C	100%	0%	0%	100%	0.119
9	Cu_2_O RD crystals	N_2_H_4_	45	50 °C	7.8%	50.2%	40.9%	8.9%	0.003
10	Cu_2_O RD crystals	NaBH_4_	45	50 °C	36.5%	90.1%	8.5%	1.4%	0.014

### Mechanism for obtaining high catalytic stereoselectivity

According to the Horiuti–Polanyi mechanism,^22^,^39^ initially DPA gets adsorbed onto the Cu surface in the presence of hydrogen atoms (Fig. S27, ESI†). In the first hydrogenation step *via* a *cis*-addition of two hydrogen atoms, the DPA molecule is converted to (*Z*)-stilbene. To confirm the source of both hydrogen atoms in the product alkene and their mode of addition, semihydrogenation of DPA was carried out under our standard conditions but by replacing anhydrous EtOH with anhydrous EtOD. As evidenced by the ^1^H-NMR spectrum (Fig. S28, ESI†), semihydrogenation of DPA in EtOD afforded the non-deuterated (*Z*)-stilbene, indicating that both hydrogen atoms come from AB and get attached *via* the *cis*-mode. Once (*Z*)-stilbene is formed on the surface, it can desorb or react further to give the unwanted formation of bibenzyl *via* a second hydrogenation step^22^ or (*E*)-stilbene by *cis*–*trans* isomerization (Fig. S27, ESI†).^39^ An efficient selective catalyst for the semihydrogenation of alkynes is characterized by having a high stability of adsorbed alkynes and a low stability of alkenes. The former defines the overall conversion of alkynes, whereas the latter measures the product selectivity. An in-depth study for the adsorption behavior of acetylene (C_2_H_2_) and ethylene (C_2_H_4_) on a Cu (111) plane has been investigated.^40^ The results clearly indicate that acetylene gets chemisorbed on the Cu {111} surface with a higher stability, whereas ethylene can be found as a weakly perturbed physisorbed species on the Cu (111) plane. In addition, there are some reports showing that alkenes have a lower binding affinity towards the Cu surface compared to alkynes, which makes Cu a highly desirable candidate for industrial gas-phase semihydrogenation reactions over Pd.^40^–^42^ In fact, continued reaction in the presence of (*Z*)-stilbene and Cu rhombic dodecahedra does not lead to additional formation of bibenzyl (Fig. S29, ESI†). So (*Z*)-stilbene should preferentially desorb from the Cu {110} surface after its formation, resulting in the highest product stereoselectivity for rhombic dodecahedral nanocrystals, and this is another example of crystal facet-dependent organocatalysis.

## Conclusions

The pseudomorphic formation of Cu rhombic dodecahedra, cubes, and octahedra at 30–50 °C from Cu_2_O crystals of corresponding shapes through simple ammonia borane reduction in ethanol has been demonstrated, opening up a new method of polyhedral metal nanocrystal formation *via* direct metal oxide conversion. Hydrogen atoms derived from ammonia borane are directly used for the Cu crystal-catalyzed stereoselective semihydrogenation of alkynes. Cu rhombic dodecahedra showed 100% conversion and an exceptional *cis*-product selectivity. The high stereoselectivity of Cu rhombic dodecahedra is likely due to their exposed {110} faces. This low-cost and highly efficient catalytic system should find broad applications.

## Experimental

### Chemicals

Anhydrous copper(ii) chloride (CuCl_2_; 97%) and hydroxylamine hydrochloride (NH_2_OH·HCl; 99%) were purchased from Aldrich. Sodium hydroxide (98.2%) and sodium dodecyl sulfate (SDS; 100%) were acquired from Mallinckrodt. Diphenylacetylene (DPA; 98%), ammonia borane (H_3_N–BH_3_; 97%), anhydrous ethanol (99.5%), ethanol-*d* (C_2_H_5_OD; 99 atom% D), Cu_2_O (99.99%), and Lindlar catalyst (5% palladium deposited on calcium carbonate poisoned with lead) were purchased from Aldrich. All chemicals were used as received without further purification. Ultrapure distilled and deionized water (18.3 MΩ) was used for all solution preparations. Commercially available reagents were used for the semihydrogenation reactions.

### Synthesis of Cu_2_O rhombic dodecahedral and cubic nanocrystals

For the large scale synthesis, 0.348 g of SDS was added to two separate 50 mL glass vials, followed by the addition of 27.80 and 35.68 mL deionized water for the synthesis of Cu_2_O rhombic dodecahedra and cubes, respectively. The sample vials were sonicated until the SDS dissolved completely and then placed in a water bath set at 31 °C. A solution of CuCl_2_ (0.1 M, 2 mL) was added to each of the vials with shaking for 1 min. For rhombic dodecahedra, NaOH (1.0 M, 0.72 mL) and NH_2_OH·HCl solutions (0.1 M, 9.48 mL) were added into the vial with a 3 s interval, then the vial was shaken for 10 s and kept in the water bath for 1 h for crystal growth. Similarly, for cubic crystals, NaOH (1.0 M, 0.72 mL) and NH_2_OH·HCl solutions (0.1 M, 0.4 mL) were added into the vial. The total volume of the final solution was 40 mL in each case. After 1 h, the reaction mixtures were centrifuged at 5000 rpm for 3 min. After decanting the top solution, the precipitate was washed with 40 mL of ethanol three times to remove unreacted chemicals and SDS surfactant. The precipitate was dispersed in 4 mL of ethanol for conversion to Cu crystals. For semihydrogenation reaction, the final precipitates were dried under high vacuum overnight.

### Synthesis of Cu_2_O octahedral nanocrystals

For the large scale synthesis, 0.348 g of SDS was added to a 50 mL glass vial, followed by the addition of 36.08 mL of deionized water. The sample vial was sonicated until the SDS dissolved completely and then placed in a water bath set at 31 °C. A solution of CuCl_2_ (0.1 M, 0.4 mL) was added to the vial with shaking for 1 min. After that, NaOH (1.0 M, 0.8 mL) and NH_2_OH·HCl solutions (0.2 M, 2.72 mL) were added into the vial with a 3 s interval, then the vial was shaken for 10 s and kept in the water bath for 2 h for crystal growth. The total volume of the final solution was 40 mL. After 2 h, the reaction mixture was centrifuged at 5000 rpm for 3 min. After decanting the top solution, the precipitate was washed with 40 mL of ethanol three times to remove unreacted chemicals and SDS surfactant. The precipitate was dispersed in 4 mL of ethanol. The final precipitate was dried under high vacuum overnight.

### Synthesis of Cu polyhedra

For the pseudomorphic formation of Cu polyhedra from Cu_2_O crystals, 0.036 g of ammonia borane was dissolved in 7.5 mL of ethanol with stirring in a 20 mL glass vial, which was kept in a water bath at 30 °C. From the above-mentioned 4 mL concentrated Cu_2_O nanocrystal solution, 2.4 mL of the solution was withdrawn and added to the ammonia borane solution with continuous stirring. For rhombic dodecahedral, cubic, and octahedral nanocrystals, the solution was stirred for 3, 5, and 7 min respectively until the solution completely changed to reddish brown. After that, stirring stopped and the particles were aged for another 2 min to allow the particles to settle down. After carefully withdrawing the top solution, the precipitate was washed with 10 mL of ethanol three times just by shaking for 2 min to remove the chemicals. The synthesized Cu polyhedra were dispersed in 1 mL of anhydrous ethanol by sonication for further characterization.

### Semihydrogenation catalysis

In a typical catalytic process, 4 mg of Cu_2_O nanocrystals (0.056 mmol Cu) and 1 mL of anhydrous ethanol were added to a 25 mL Ace pressure tube and sonicated for 1 min to disperse the catalyst homogeneously in the solution. 1 mL of anhydrous ethanolic solution of diphenylacetylene (0.0178 g, 0.1 mmol) and 1 mL of anhydrous ethanolic solution of ammonia borane (0.0123 g, 0.4 mmol) were added one by one and then sealed in the tube. The Ace pressure tube was placed in a water bath kept at 50 °C with continuous stirring. After 3 min, the solution color changed to reddish brown confirming the *in situ* pseudomorphic formation of Cu crystals, which then acted as the active catalyst for the semihydrogenation of alkynes. After completion of the reaction, the clear top anhydrous ethanolic reaction mixture was carefully collected, leaving the Cu crystals behind, and the solvent was removed under reduced pressure. 4 mL of dichloromethane was added to extract the products and subjected to evaporation to obtain the crude compound, which was directly used for ^1^H-NMR analysis.

Time-dependent studies for the conversion of DPA and selectivity of different products catalyzed by Cu RDs, cubes, and octahedra were also done by collecting the ^1^H-NMR spectrum of the crude isolated product. In all cases, 4 mg of Cu_2_O nanocrystals was used, keeping a fixed Cu molar mass (*i.e.* 0.056 mmol). We further used CuCl_2_ (7.5 mg, 0.056 mmol Cu), commercial Cu_2_O (4 mg, 0.056 mmol Cu), and commercial Lindlar catalyst (121.7 mg, 0.056 mmol Pd) under the same reaction conditions to evaluate their catalytic activities and product selectivity relative to that of Cu rhombic dodecahedra. For the Lindlar catalyst, we used hydrogen gas in the presence of 100 μL of quinoline. When ammonia borane was used as a source of hydrogen, no quinoline was added. To evaluate the general applicability, semihydrogenation of the internal alkyne was performed using different alkynes. Cu rhombic dodecahedra were chosen as the catalyst.

### Deuterium labelling experiments

Under the protection of nitrogen gas, 4 mg of Cu_2_O rhombic dodecahedra and 1 mL of ethanol-*d* (C_2_H_5_OD) were added to a 25 mL Schlenck tube equipped with a magnetic stirrer bar, and sonicated for 1 min to disperse the catalyst homogeneously in the solution. Next, 1 mL of ethanol-*d* solution of diphenylacetylene (0.0178 g, 0.1 mmol) and 1 mL of ethanol-*d* solution of ammonia borane (0.0123 g, 0.4 mmol) were added. The Schlenck tube was then placed in a water bath kept at 50 °C with continuous stirring. After completion of the reaction, the clear top ethanol-*d* solution was carefully collected and the solvent was removed under reduced pressure. 4 mL of dichloromethane was added to extract the products and they were subjected to evaporation to obtain the crude compound, which was directly used for ^1^H-NMR analysis.

### Characterization

SEM images of the samples were obtained using a JEOL JSM-7000F electron microscope. TEM characterization was performed on a JEOL JEM-2100 microscope with an operating voltage of 200 kV. Elemental mapping images were acquired by EDS using the same JEOL JEM-2100 electron microscope equipped with a STEM unit and an Inca Energy 250 detector from Oxford Instruments. XPS characterization was carried out on a ULVAC-PHI Quantera SXM high-resolution XPS spectrometer. A monochromatized Al anode was used as the excitation source. The C 1s peak was chosen as the reference line. XRD patterns were recorded on a Shimadzu XRD-6000 diffractometer with Cu Kα radiation. UV-vis absorption spectra were taken using a JASCO V-670 spectrophotometer.

## Conflicts of interest

There are no conflicts to declare.

## Supplementary Material

Supplementary information
