# Supplementary material for: Polyhedral Cu2O to Cu pseudomorphic conversion for stereoselective alkyne semihydrogenation
Source: Chem Sci. 2018 Jan 31;9(9):2517–24. doi: 10.1039/c7sc05232d (PMC5911822; doi:10.1039/c7sc05232d)
Supplement: Supplementary file 1 [file SC-009-C7SC05232D-s001.pdf]

Electronic Supplementary Material (ESI) for Chemical Science.
This journal is © The Royal Society of Chemistry 2018

Electronic Supplementary Information

Polyhedral Cu_2O to Cu pseudomorphic conversion for stereoselective alkyne semihydrogenation

Sourav Rej,‡ Mahesh Madasu,‡ Chih-Shan Tan, Chi-Fu Hsia, and Michael H. Huang*
Department of Chemistry, National Tsing Hua University, Hsinchu 30013, Taiwan.
‡ These authors contributed equally to this work.

Experimental

Synthesis of Cu_2O rhombic dodecahedral and cubic nanocrystals

For the large scale synthesis, 0.348 g of SDS was added in two separate 50-mL glass vials, followed by the addition of 27.80 and 35.68 mL deionized water for the synthesis of Cu_2O rhombic dodecahedra and cubes, respectively. The sample vials were sonicated until SDS was dissolved completely and then placed in a water bath set at 31 ºC. A solution of $CuCl_2$ (0.1 M, 2 mL) was added to each of the vials with shaking for 1 min. For rhombic dodecahedra, NaOH (1.0 M, 0.72 mL) and $NH_2OH{\cdot}HCl$ solutions (0.1 M, 9.48 mL) were added into the vial with 3 s interval, then shook for 10 s and kept in the water bath for 1 h for crystal growth. Similarly, for cubic crystals, NaOH (1.0 M, 0.72 mL) and $NH_2OH{\cdot}HCl$ solutions (0.1 M, 0.4 mL) were added into the vial. The total volume of the final solution is 40 mL in each case. After 1 h, the reaction mixtures were centrifuged at 5000 rpm for 3 min. After decanting the top solution, the precipitate was washed with 40 mL of ethanol for three times to remove unreacted chemicals and SDS surfactant. The precipitate was dispersed in 4 mL of ethanol for conversion to Cu crystals. For semihydrogenation reaction, the final precipitates were dried under high vacuum for overnight.

Synthesis of Cu_2O octahedral nanocrystals

For the large scale synthesis, 0.348 g of SDS was added to a 50-mL glass vial, followed by the addition of 36.08 mL of deionized water. The sample vial was sonicated until SDS became dissolved completely and then placed in a water bath set at 31 ºC. A solution of $CuCl_2$ (0.1 M, 0.4 mL) was added to the vial with shaking for 1 min. After that, NaOH (1.0 M, 0.8 mL) and $NH_2OH{\cdot}HCl$ solutions (0.2 M, 2.72 mL) were added into the vial with 3 s interval, then shook for 10 s and kept in the water bath for 2 h for crystal growth. The total volume of the final solution is 40 mL. After 2 h, the reaction mixture was centrifuged at 5000 rpm for 3 min. After decanting the top solution, the precipitate was washed with 40 mL of ethanol for three times to remove unreacted chemicals and SDS surfactant. The precipitate was dispersed in 4 mL of ethanol. The final precipitate was dried under high vacuum for overnight.

TOF calculations

TOF = (No. of mmol of product formed)/(No. of mmol of metal catalyst × time)

Since 0.1 mmol of starting DPA was used, 100% conversion should yield 0.1 mmol of product. No. of mmol of metal (usually Cu) present in each catalyst = 0.056 mmol

TOF of Cu_2O RD crystals (entry 1 in Table 1) = 0.1 mmol/(0.056 mmol × 45 min) = 0.039 min^{-1}

For Cu_2O cubes (entry 2 in Table 1), 95.6% conversion means 0.0956 mmol of product has been formed.

TOF = 0.0956 mmol/(0.056 mmol × 60 min) = 0.028 min^{-1}

TOF numbers for other catalysts were calculated the same way.

Table S1. Cu polyhedra synthesis. The exact condition used for the synthesis of Cu RDs, cubes, and octahedra in a water bath set at 30 ºC.

Ammonia Borane (AB) ($H_3N–BH_3$)	Cu_2O Nanocrystals (40 ml reaction product concentrated in 4 ml EtOH)	Stirring	Aging
0.0360 g in 7.5 mL EtOH	2.4 mL	3 min for RD<br>5 min for Cube<br>7 min for Octahedra	2 min

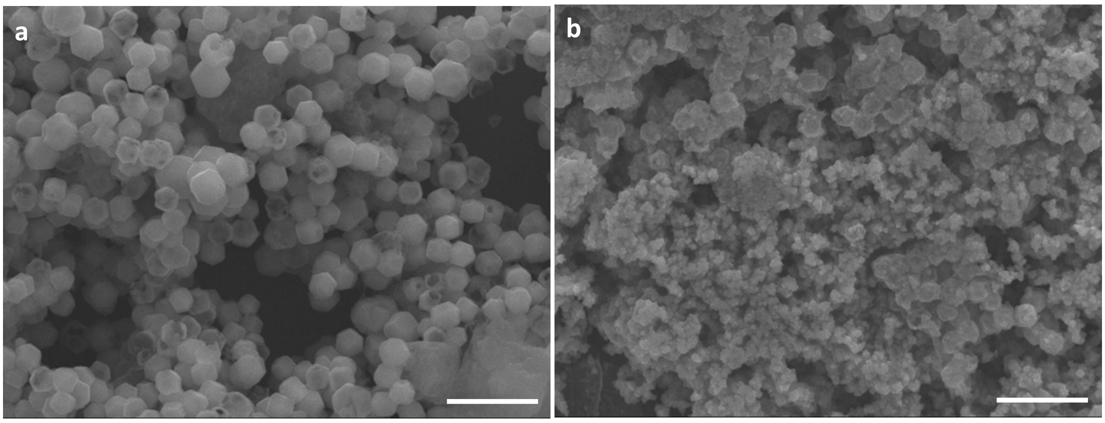

Fig. S1. The importance of ammonia borane over other reducing agents. Representative large-area SEM images of the as-synthesized Cu particles obtained by the reduction of Cu_2O RD crystals with (a) hydrazine and (b) sodium borohydride. Only when ammonia borane acts as the reducing agents for the pseudomorphic conversion of Cu_2O crystals to Cu crystals can the particle shapes be maintained. All scale bars equal to 1 μm.

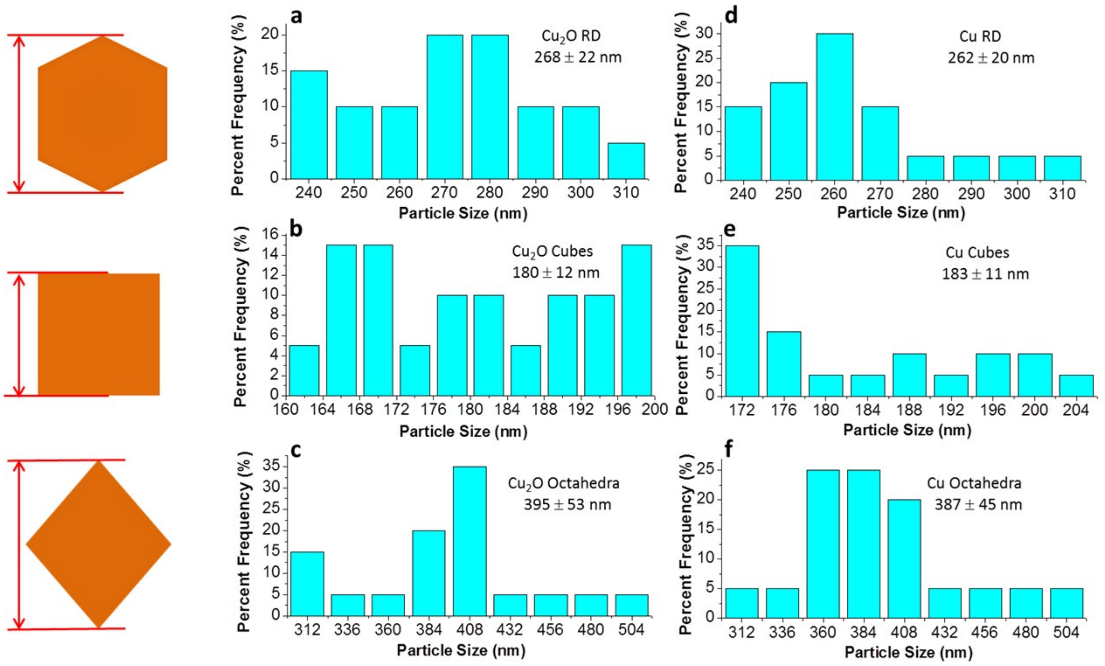

Fig. S2. Cu_2O and Cu particle sizes. Representative size distribution histograms of the Cu_2O (a) RD, (b) cubic, and (c) octahedral crystals. The size distribution histogram of the as-synthesized Cu (d) RD, (e) cubes, and (f) octahedra confirm their retention of original Cu_2O particle sizes.

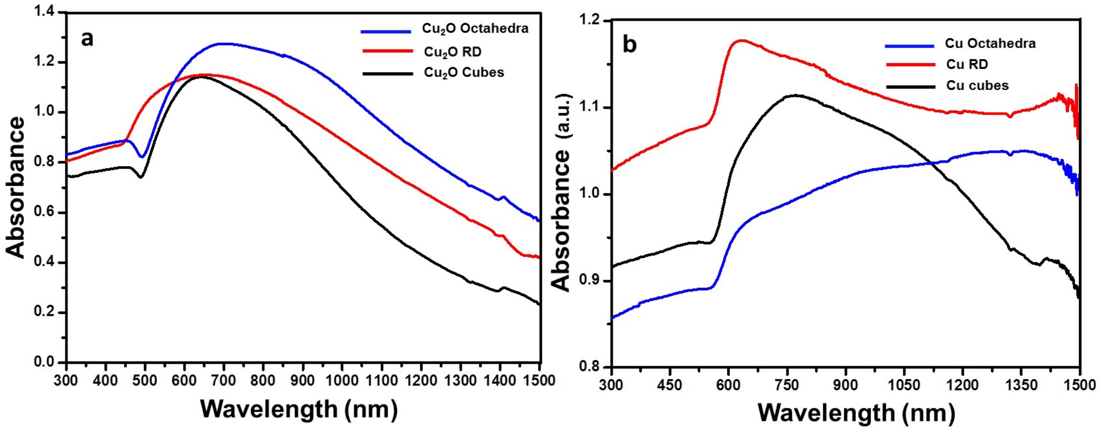

Fig. S3. UV–visible spectra of Cu_2O and Cu crystals. Representative UV–visible spectra of (a) Cu_2O and (b) Cu crystals in anhydrous ethanol for rhombic dodecahedral, cubic, and octahedral shapes.

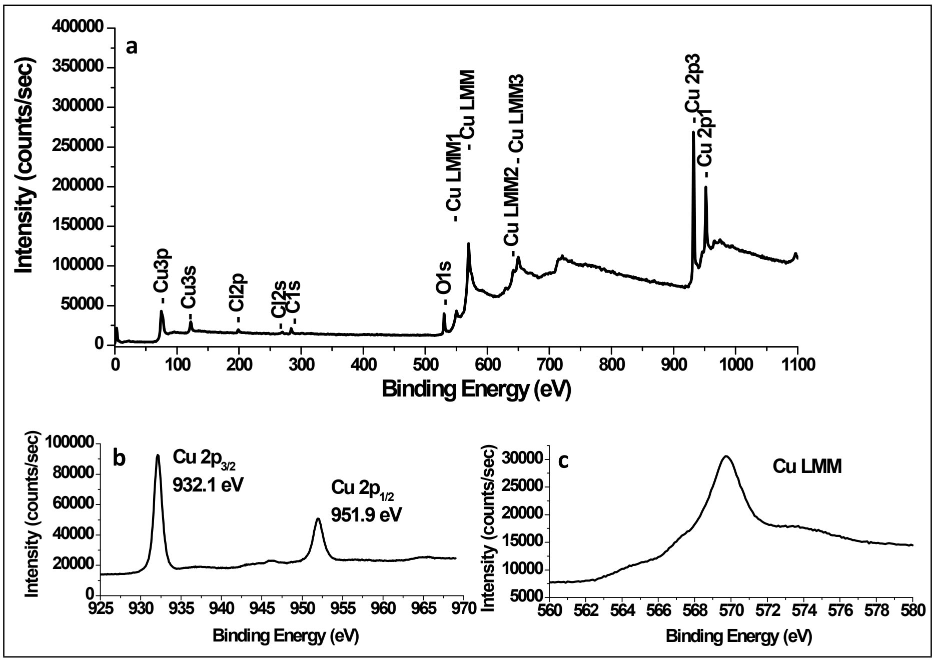

Fig. S4. X-ray photoelectron spectroscopic analysis of Cu_2O RD nanocrystals. (a) Representative full survey XPS spectrum, (b) HR-XPS showing the Cu 2p peaks, and (c) HR-XPS of the Cu LMM region for Cu_2O rhombic dodecahedra.

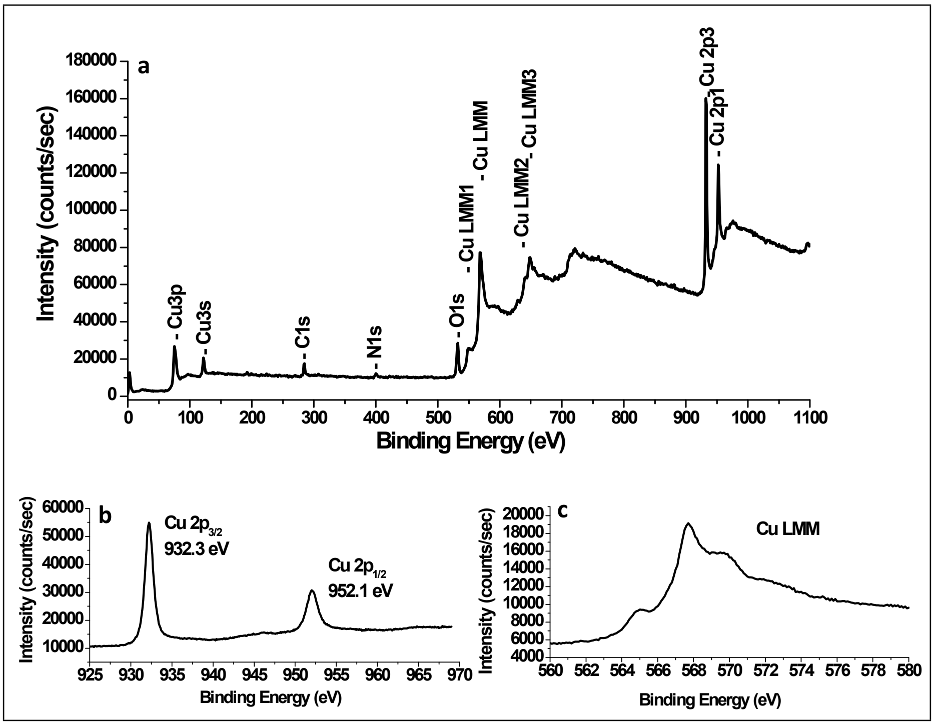

Fig. S5. X-ray photoelectron spectroscopic analysis of Cu rhombic dodecahedra. (a) Representative full survey XPS spectrum, (b) HR-XPS of the Cu 2p peaks, and (c) HR-XPS of the Cu LMM region of Cu rhombic dodecahedra. The Cu LMM peak obtained from Cu rhombic dodecahedra looks distinctively different from that of Cu_2O crystals.

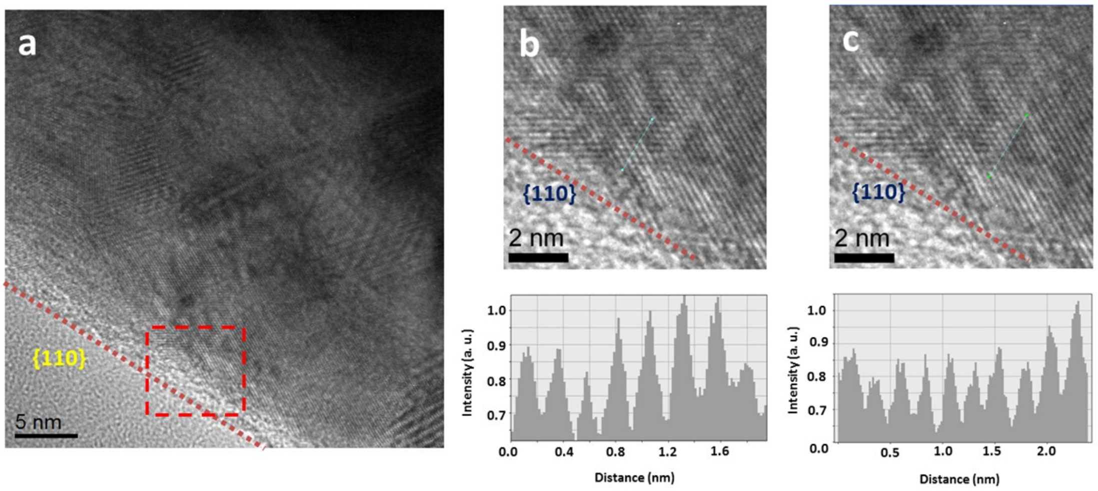

Fig. S6. Surface analysis of a Cu RD particle. (a) Large-area HR-TEM image of a Cu RD particle. The purple dash line indicates the particle edge terminated with {110} surface. (b, c) HR-TEM image of the red dash frame region in panel a for *d*-spacing analysis. A line drawn perpendicular to the {110} surface of the RD particle shows lattice planes with average *d*-spacings of 2.59 Å in panel b and 2.54 Å in panel c. The Cu (110) planes should have a *d*-spacing of 2.56 Å (twice the *d*-spacing for Cu (110) planes).

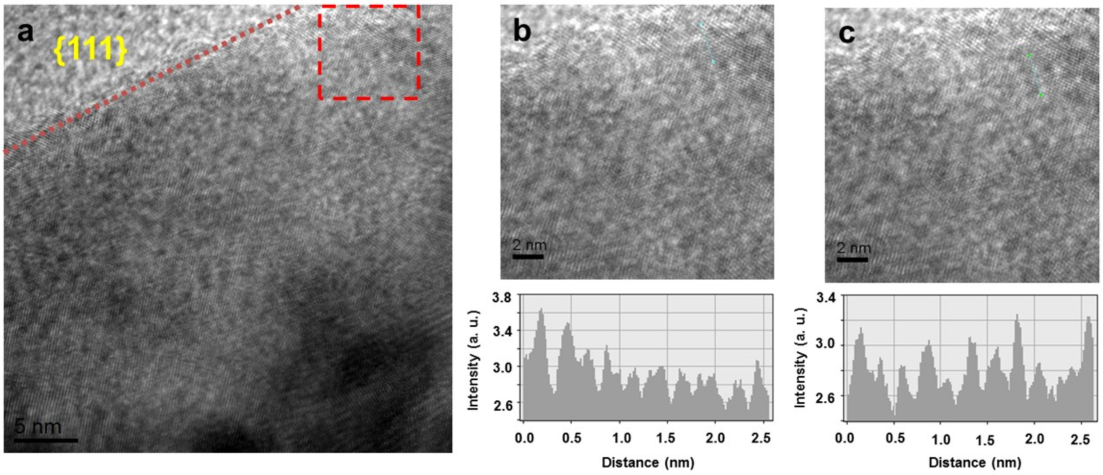

Fig. S7. Surface analysis of a Cu octahedron. (a) Large-area HR-TEM image of a Cu octahedron. The blue dash line indicates the particle edge terminated with {111} surface. (b, c) HR-TEM image of the yellow dash frame region in panel a for *d*-spacing analysis. A line drawn perpendicular to the {111} surface of the octahedron shows lattice planes with average *d*-spacings of 2.04 Å in panel b and 2.02 Å in panel c. The Cu (111) planes should have a *d*-spacing of 2.09 Å.

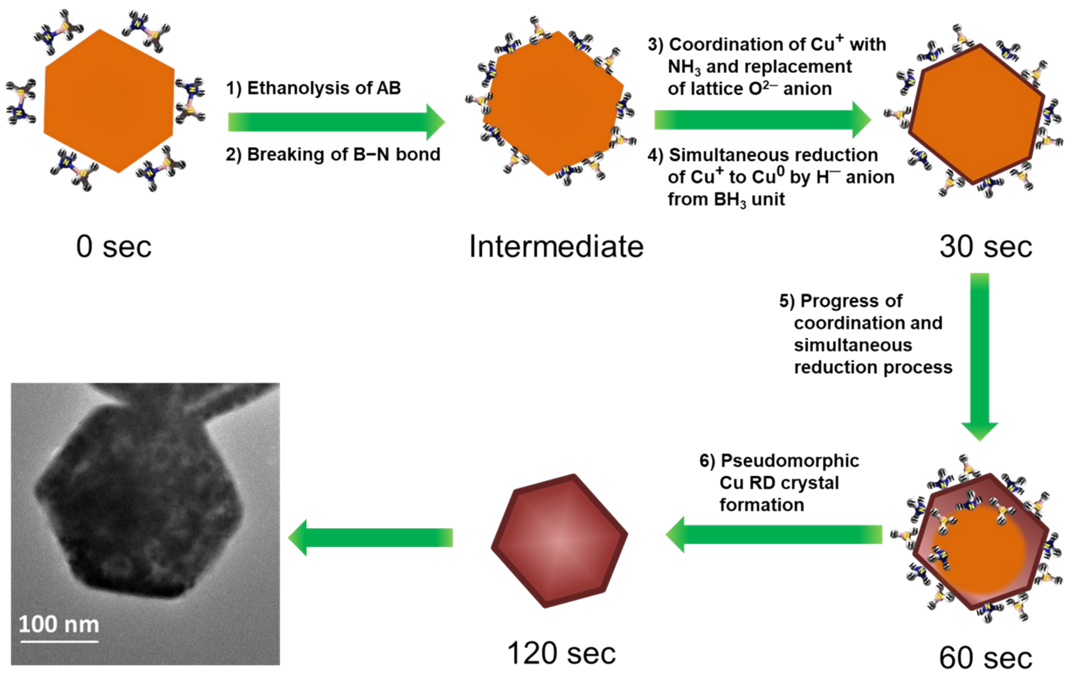

Fig. S8. Schematic diagram for the pseudomorphic formation of rhombic dodecahedral Cu crystals from Cu_2O crystals. Orange and reddish-brown colors represent Cu_2O and Cu, respectively. Ammonia borane ($H_3N–BH_3$) is used as a selective reducing agent for the pseudomorphic reduction of Cu_2O to Cu crystals.

When an ethanolic solution of AB is added to the Cu_2O crystal suspension, the protonic hydrogens of NH_3 unit in AB prefer to attach on the surface O^{2-} anions, whereas the hydridic hydrogens of BH_3 unit prefer the surface Cu^+ cations of Cu_2O crystals. Such surface charge heterogeneity on the Cu_2O crystals enhances the binding of AB, which in turn effectively reduces the activation barrier and weakens the B–N bond in AB. Ethanol molecules subsequently attack on these weakened “B–N” bonds to generate free BH_3 and NH_3 intermediates (Equation 1). According to the Pearson’s hard and soft acid–base (HSAB) principle, soft–soft interaction of $Cu^+–NH_3$ is much stronger than the soft–hard interaction of $Cu^+–O^{2-}$ within the crystal, which allows a stronger coordination of NH_3 molecules with Cu^+ cations present on the crystal surface, forming $Cu^+–NH_3$ bonds.[1] This process leads to the replacement of lattice O^{2-} anions. When this coordination process occurs, nearby free BH_3 molecules undergo ethanolysis and releases hydride (H^-) anions (Equation 2), which synchronously reduces $Cu^+–NH_3$ unit to Cu^0 metal with liberating NH_3 (Equation 3) and hydrogen gas molecules (Equation 4).

$$H_3N–BH_3 + EtOH \rightarrow NH_4^+ + [BH_3(OEt)]^- \leftrightarrow NH_3 + BH_3 + EtOH \quad (1)$$

$$BH_3 + 3EtOH \rightarrow B(OEt)_3 + 3H^+ + 3H^- \quad (2)$$

$$Cu^+–NH_3 + H^- \rightarrow Cu^0–H + NH_3\uparrow \quad (3)$$

$$2Cu^0–H \rightarrow 2Cu + H_2\uparrow \quad (4)$$

References:

1. J. Nai, Y. Tian, X. Guan and L. Guo, *J. Am. Chem. Soc.*, 2013, **135**, 16082–16091.

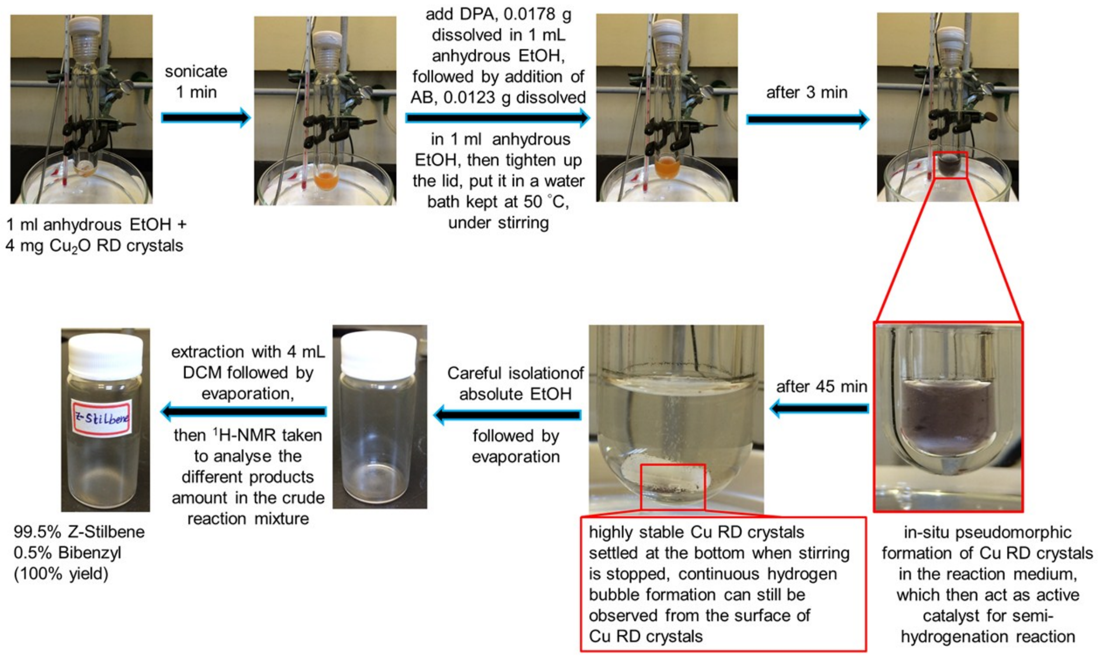

Fig. S9. Representative photographs for the highly reproducible, green, low cost, hydrogen economic and one-pot stereoselective semihydrogenation of DPA to (*Z*)-stilbene using rhombic dodecahedral Cu_2O crystals and anhydrous ethanolic solution of ammonia borane via in-situ pseudomorphic formation of Cu rhombic dodecahedra within 3 minutes after starting the reaction. This can be visualized by the color changes from orange for Cu_2O particles to reddish brown Cu particles. Isolation and purification of the products is also shown. No further column chromatography is needed for the purification of (*Z*)-stilbene. To quantify different products, direct ^{1}H-NMR spectra of the crude product were taken.

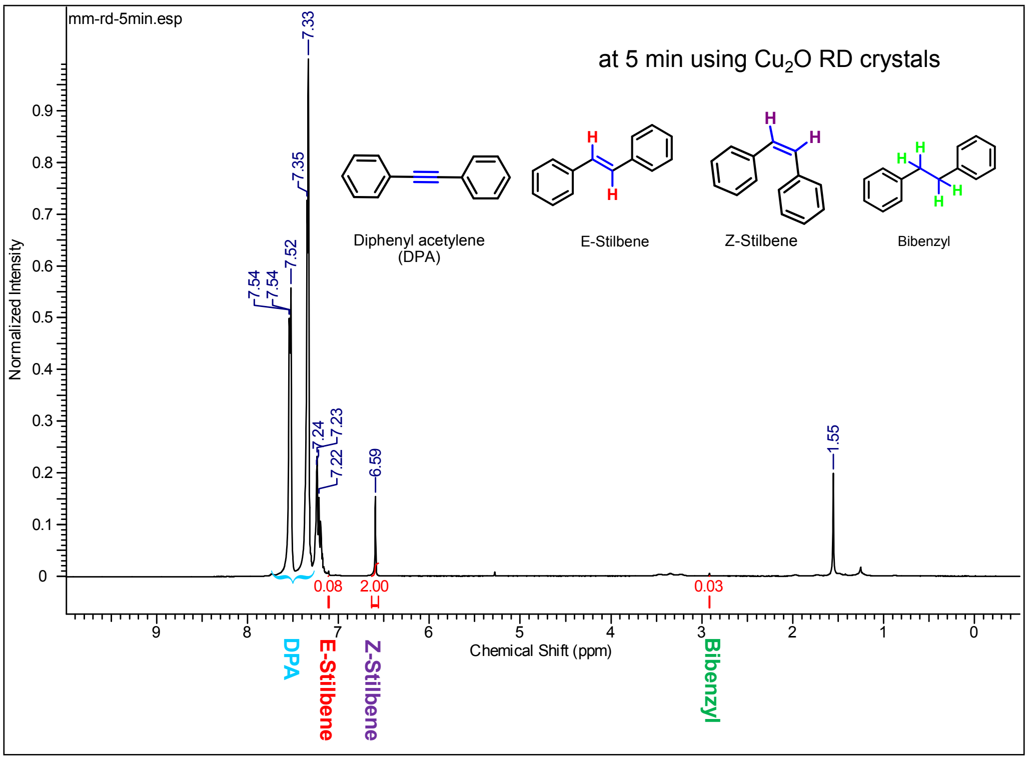
mm-rd-5min.esp
at 5 min using Cu_2O RD crystals
Diphenyl acetylene (DPA)
E-Stilbene
Z-Stilbene
Bibenzyl
Normalized Intensity
Chemical Shift (ppm)
DPA
E-Stilbene
Z-Stilbene
Bibenzyl
7.54
7.54
7.52
7.35
7.33
7.24
7.23
7.22
6.59
1.55
0.08
2.00
0.03

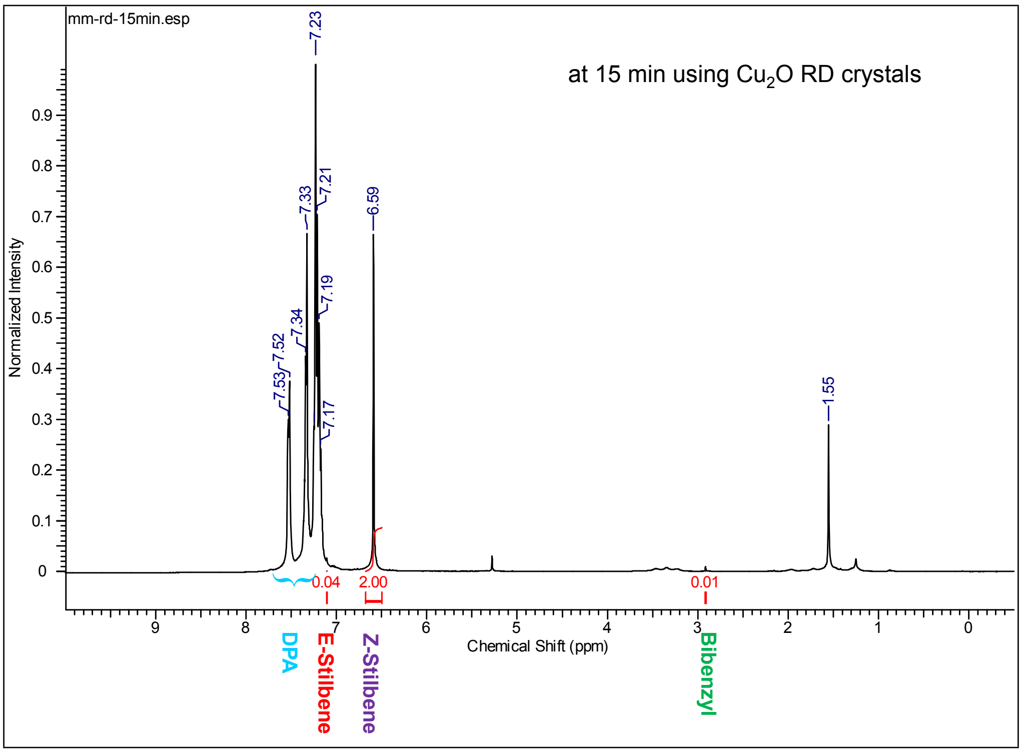
mm-rd-15min.esp
at 15 min using Cu_2O RD crystals
Normalized Intensity
Chemical Shift (ppm)
DPA
E-Stilbene
Z-Stilbene
Bibenzyl
7.53
7.52
7.34
7.33
7.23
7.21
7.19
7.17
6.59
1.55
0.04
2.00
0.01

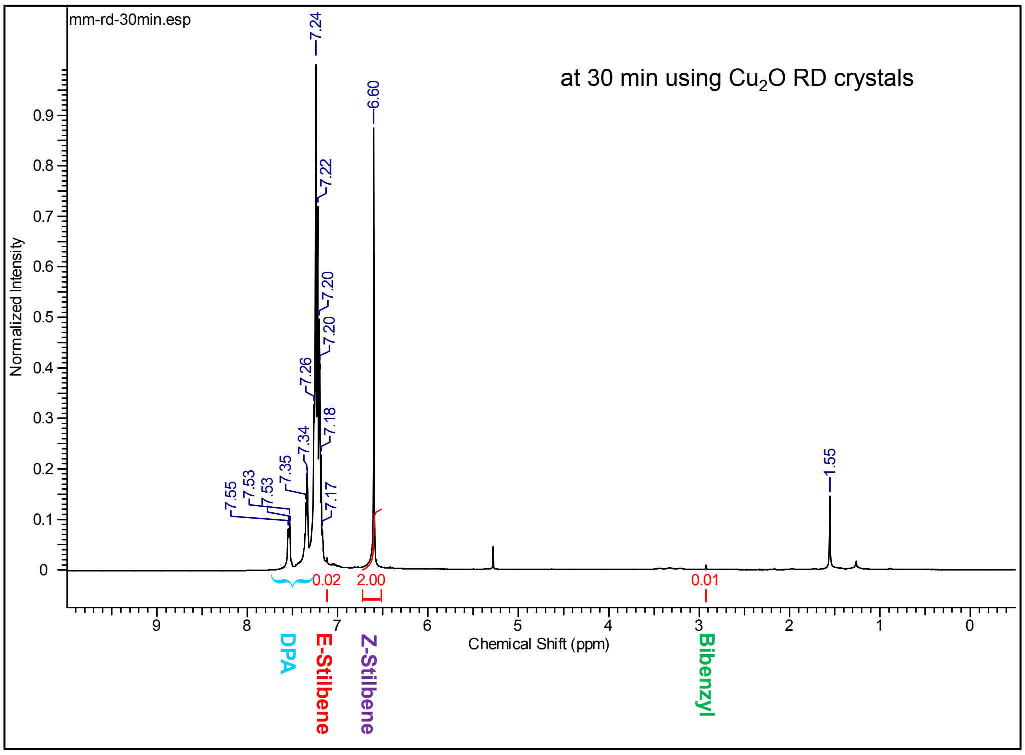

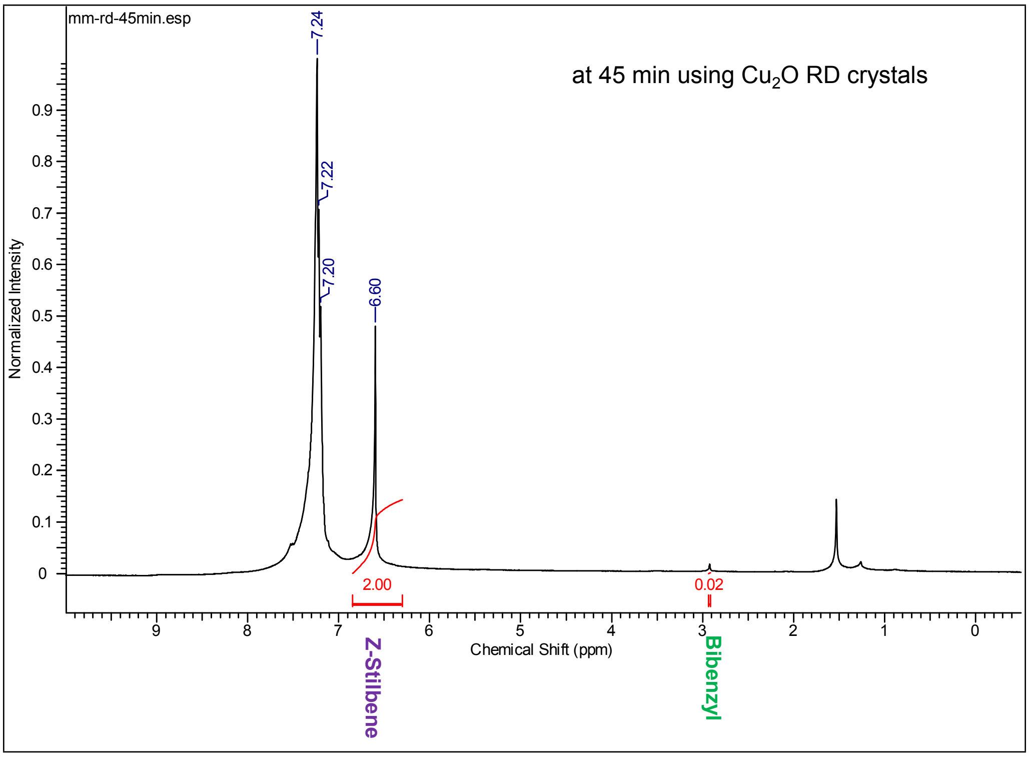

Fig. S10. Time-dependent product selectivity of Cu RD crystals. Time-dependent ^{1}H-NMR study for semihydrogenation of DPA using Cu RD crystals. No column chromatography was performed.

Table S2. Effect of the amount of ammonia borane used in the semihydrogenation of DPA keeping other parameters fixed.

Entry	NH_3BH_3 (mmol)	Catalyst (mg)	Temp / Time	EtOH (mL)	Conversion of DPA
1	0.1	Cu_2O RD (4)	50 °C / 45 min	3	40.4%
2	0.2				54.4%
3	0.3				91.3%
4	0.4				99.5%

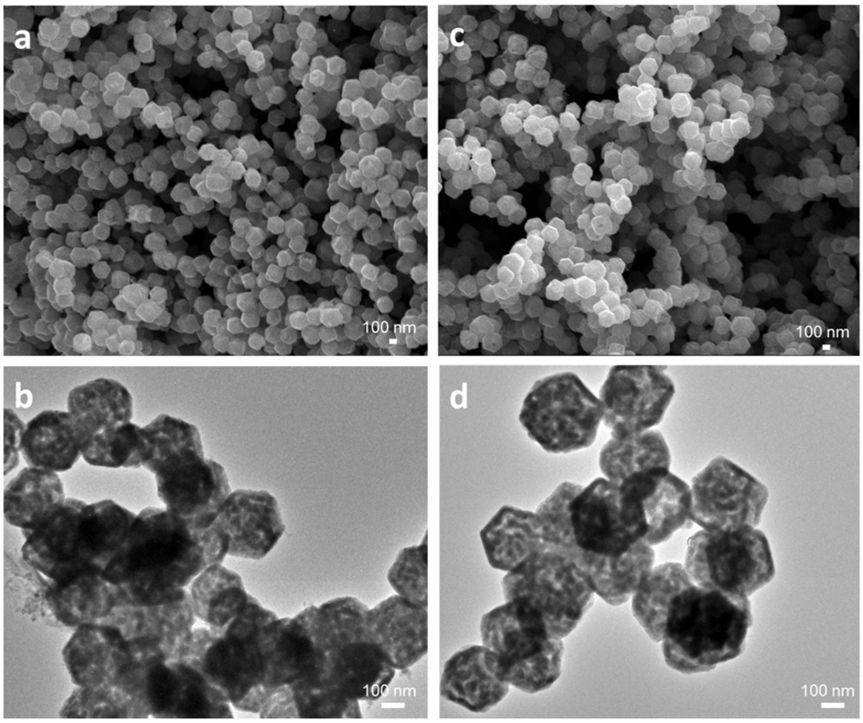

Fig. S11. SEM and TEM images for the in-situ generated Cu rhombic dodecahedra after (a, b) 3 min and (c, d) 45 min of the catalytic reaction. The images show that the rhombic dodecahedra maintain their size and shape after completion of the reaction.

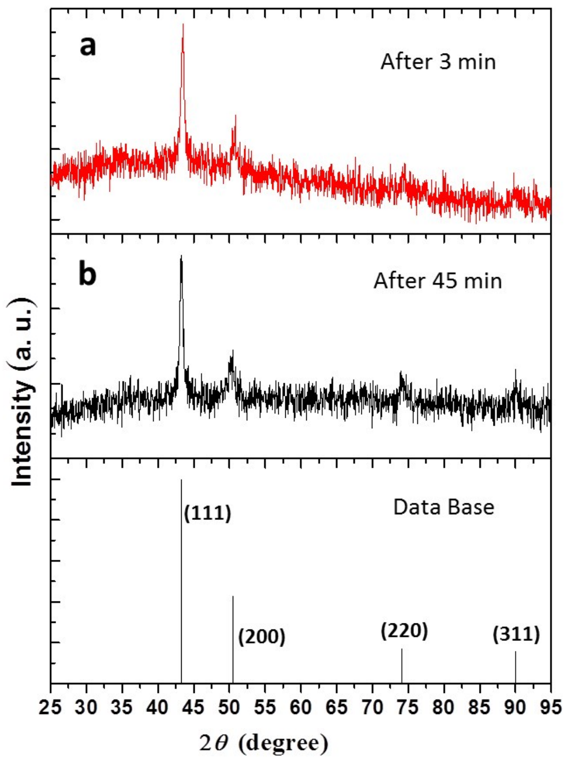

Fig. S12. Representative XRD patterns for the in-situ generated Cu rhombic dodecahedra after (a) 3 min and (b) 45 min of catalytic reaction.

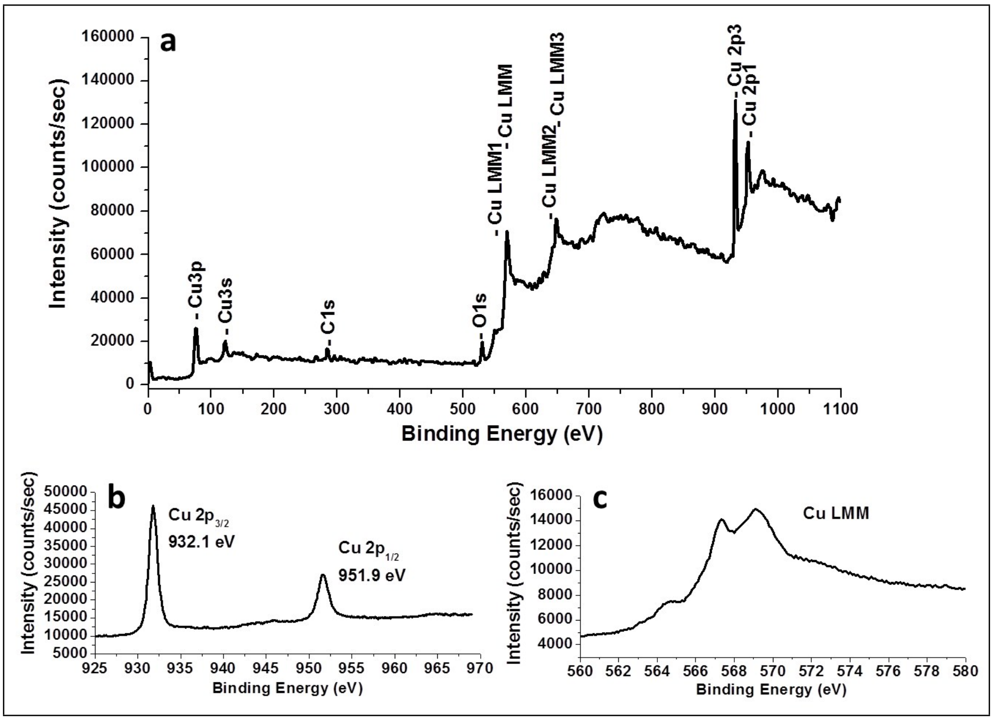

Fig. S13. X-ray photoelectron spectroscopic analysis of in-situ generated Cu rhombic dodecahedra after 3 min of the catalytic reaction. (a) Representative XPS full survey spectrum, (b) HR-XPS of the Cu 2p peak region, and (c) HR-XPS of the Cu LMM region.

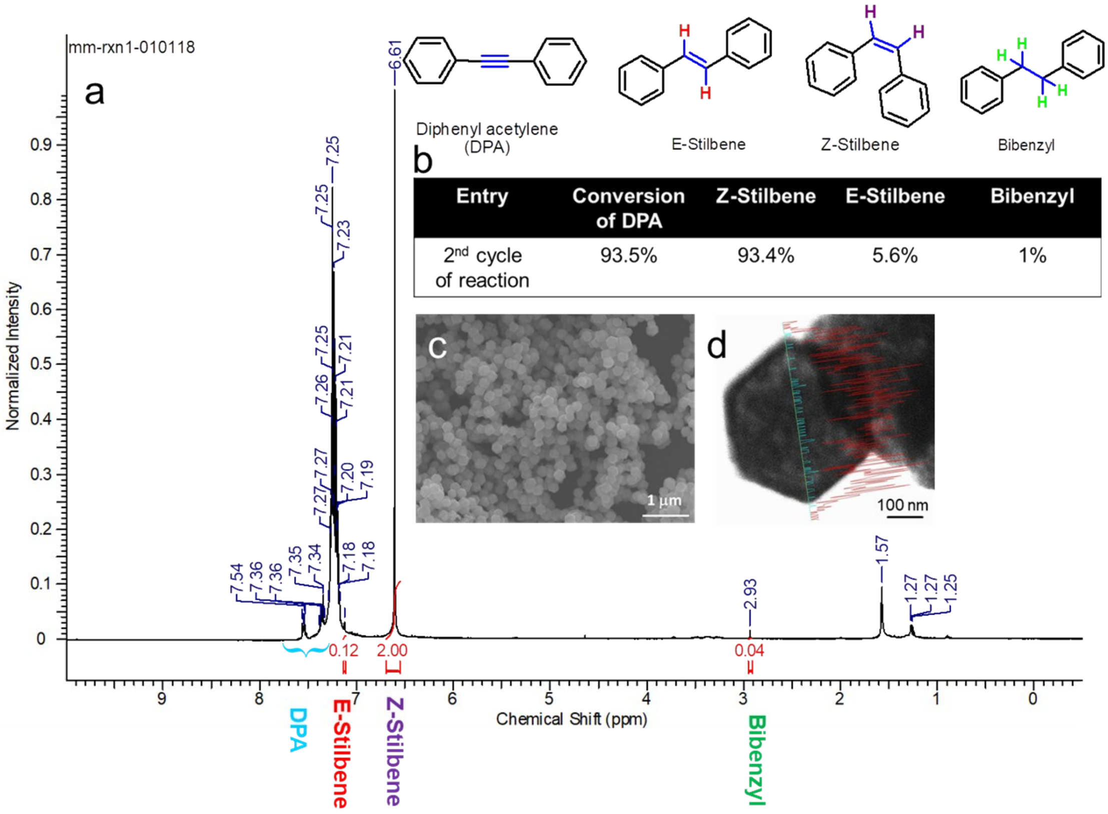

Fig. S14. Test of catalytic recyclability. (a) ^{1}H-NMR spectrum for the second cycle of the semihydrogenation of DPA using the recycled Cu RD crystals. No column chromatography was performed. (b) Coversion of DPA and product selectivity for the second cycle of the reaction. (c) SEM image of the Cu RD crystals after the second catalytic cycle. Cu RD crystals still keep their original shape. (d) HAABF-STEM image with EDS elemental line scans for a Cu RD crystal after the second cycle of the reaction. The red and blue lines correspond to copper and oxygen, respectively.

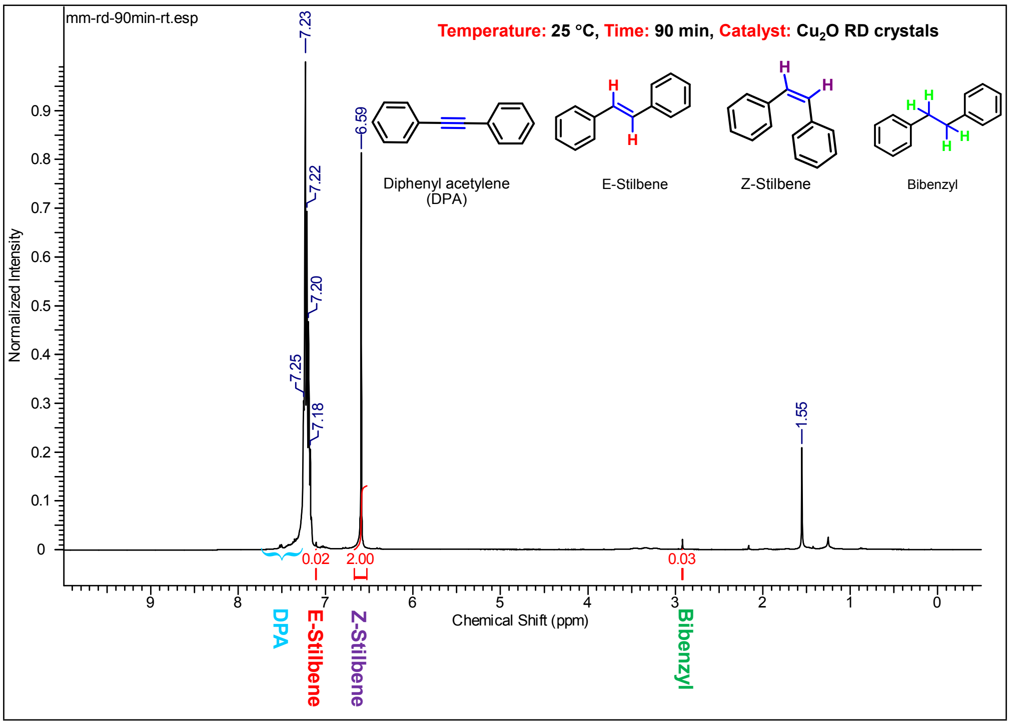

Fig. S15. ^{1}H-NMR spectrum for semihydrogenation of DPA using Cu RD crystals at room temperature. No column chromatography was performed.

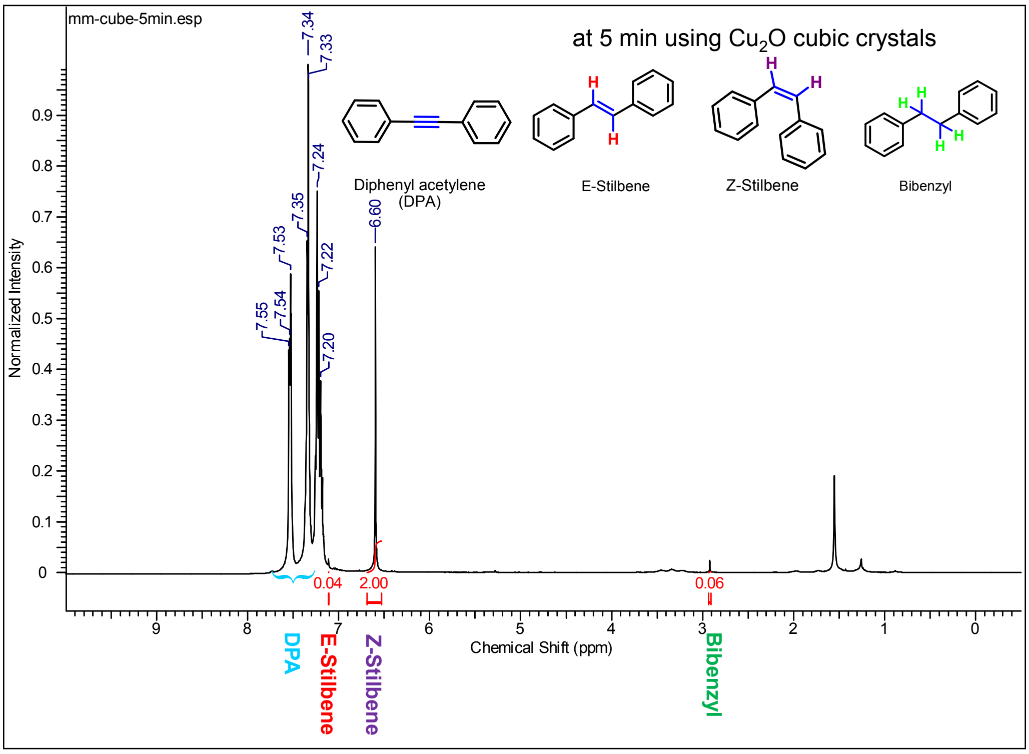

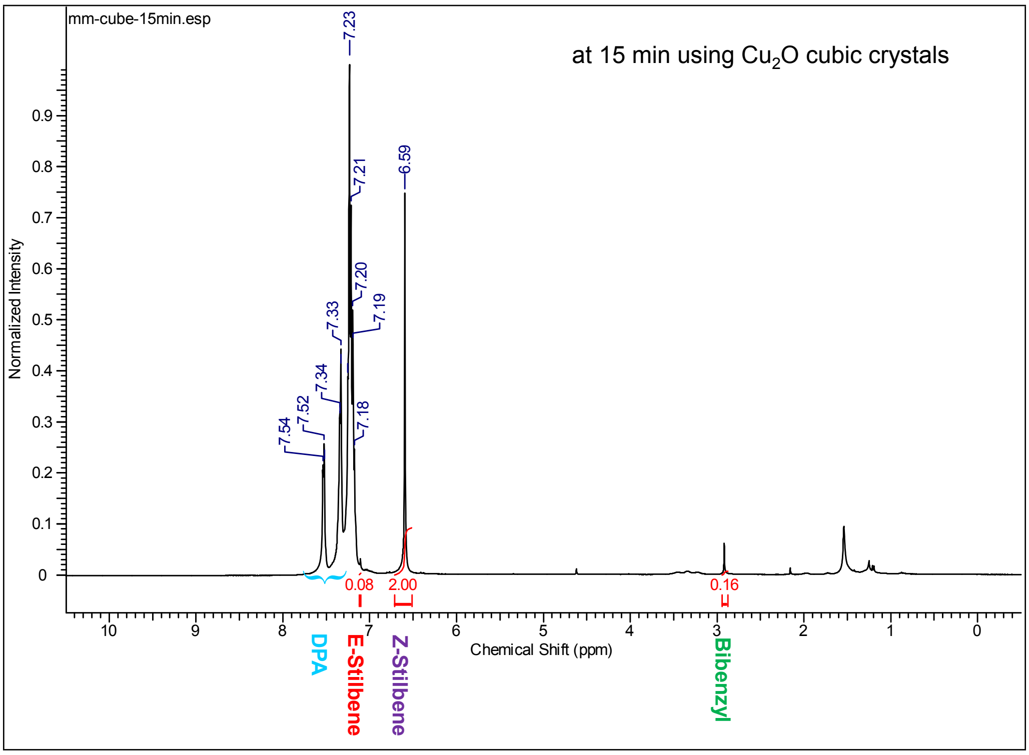
mm-cube-15min.esp
at 15 min using Cu_2O cubic crystals
Normalized Intensity
Chemical Shift (ppm)
7.23
7.21
7.20
7.19
7.33
7.34
7.52
7.54
7.18
6.59
0.08
2.00
0.16
DPA
E-Stilbene
Z-Stilbene
Bibenzyl

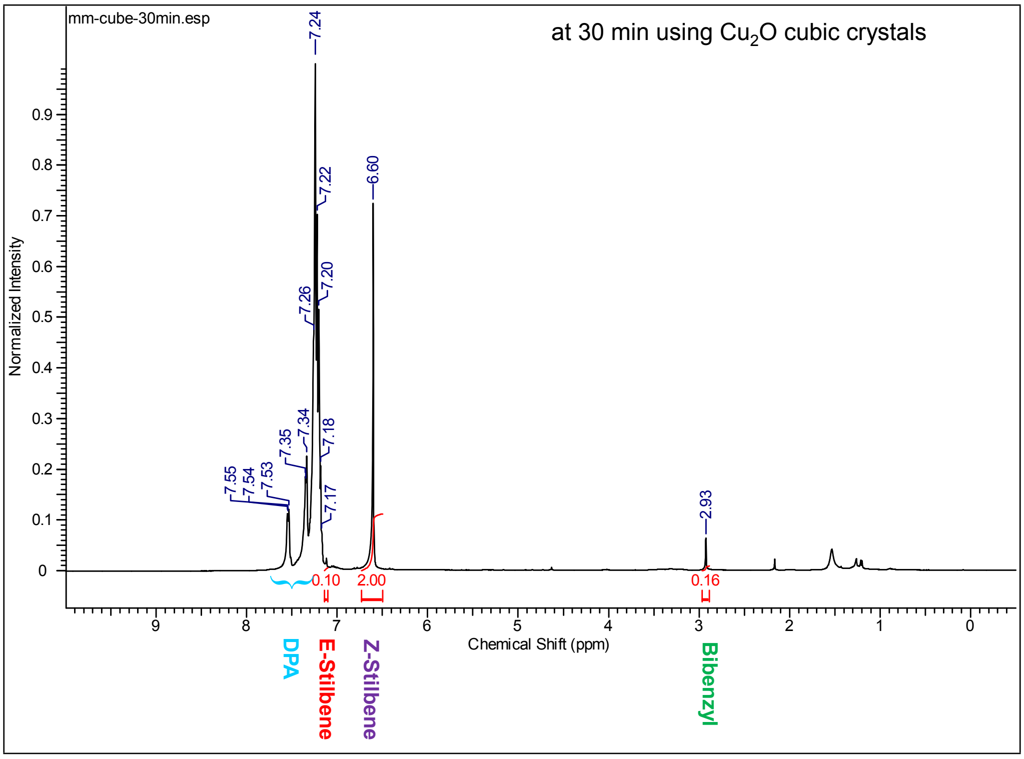
mm-cube-30min.esp
at 30 min using Cu_2O cubic crystals
Normalized Intensity
Chemical Shift (ppm)
7.24
7.22
7.20
7.26
7.35
7.34
7.18
7.55
7.54
7.53
7.17
6.60
2.93
0.10
2.00
0.16
DPA
E-Stilbene
Z-Stilbene
Bibenzyl

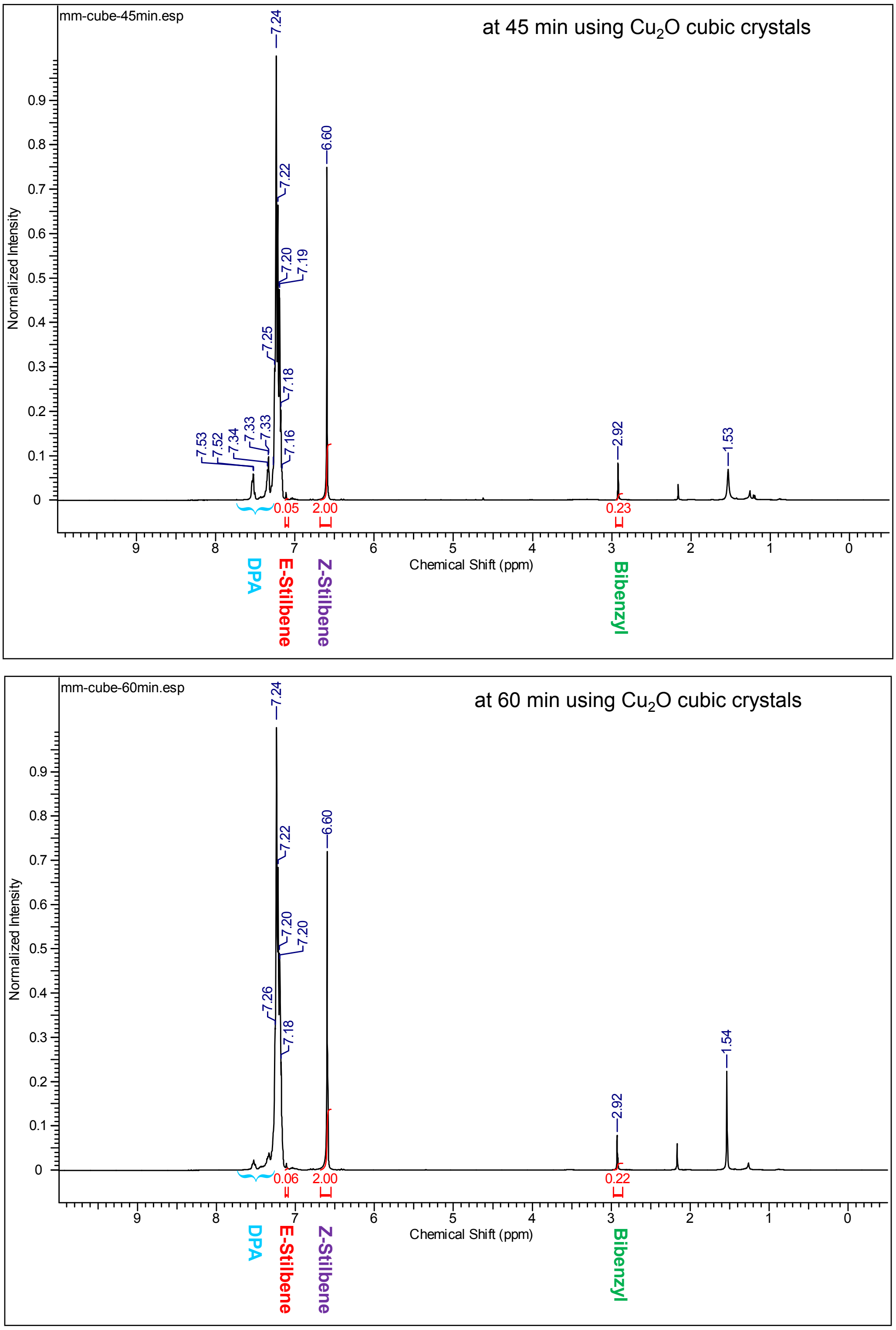

Fig. S16. Product selectivity of Cu cubes. Time-dependent ^{1}H-NMR study for semihydrogenation of DPA using Cu cubes. No column chromatography was performed.

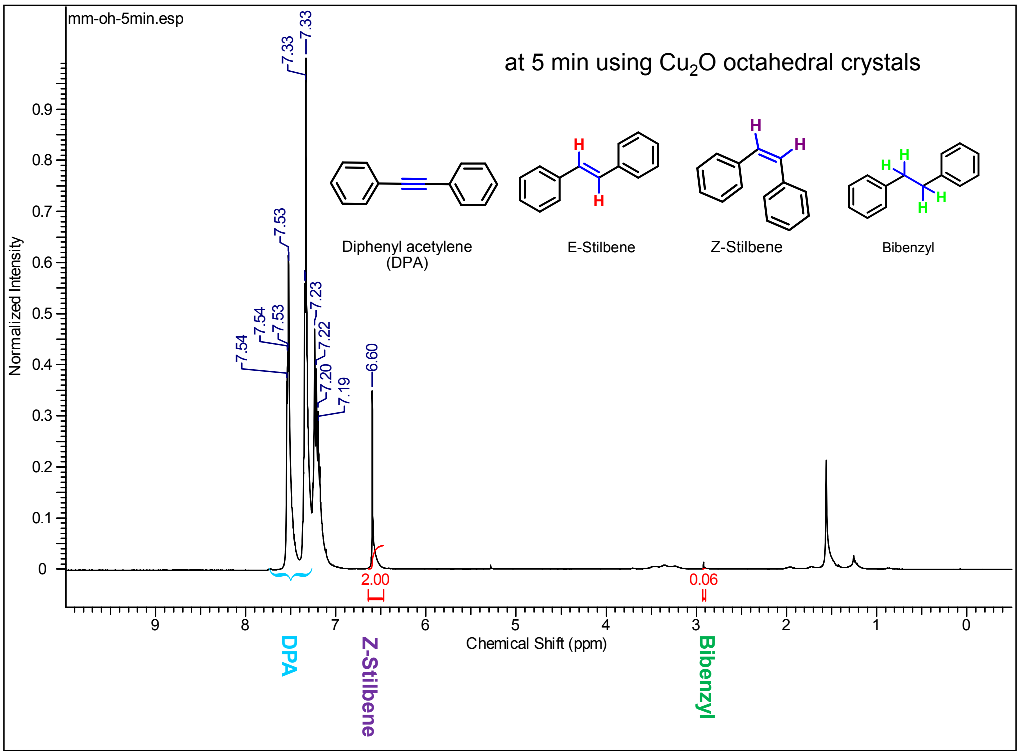
mm-oh-5min.esp
at 5 min using Cu_2O octahedral crystals
Diphenyl acetylene (DPA)
E-Stilbene
Z-Stilbene
Bibenzyl
Normalized Intensity
Chemical Shift (ppm)
DPA
Z-Stilbene
Bibenzyl
2.00
0.06

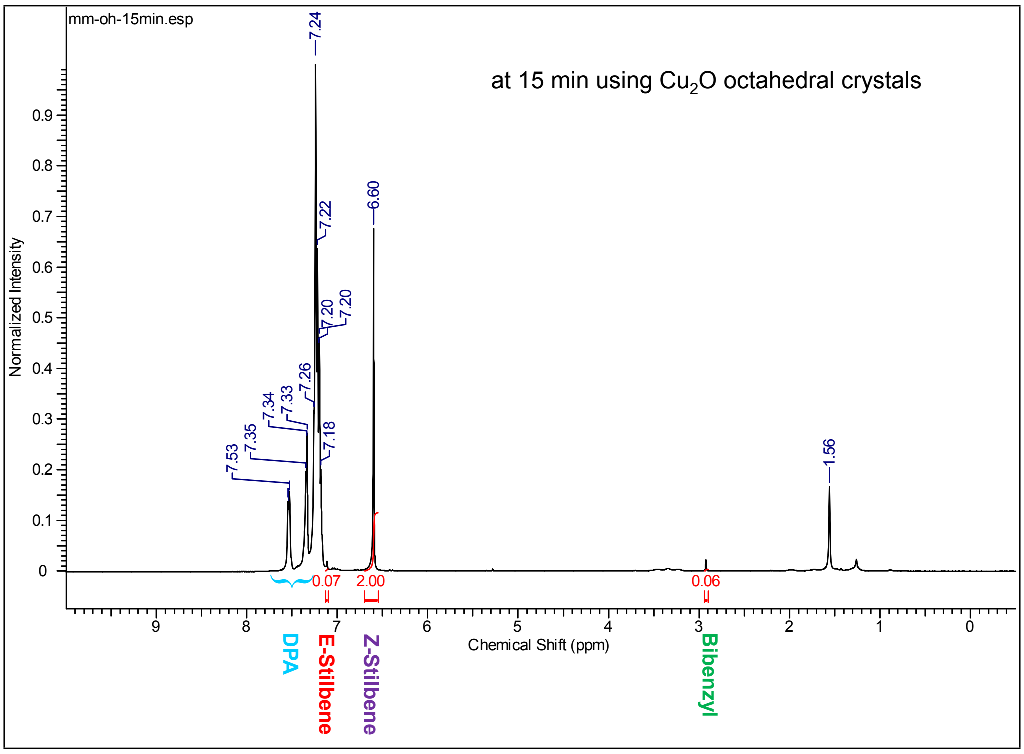
mm-oh-15min.esp
at 15 min using Cu_2O octahedral crystals
Normalized Intensity
Chemical Shift (ppm)
DPA
E-Stilbene
Z-Stilbene
Bibenzyl
0.07
2.00
0.06

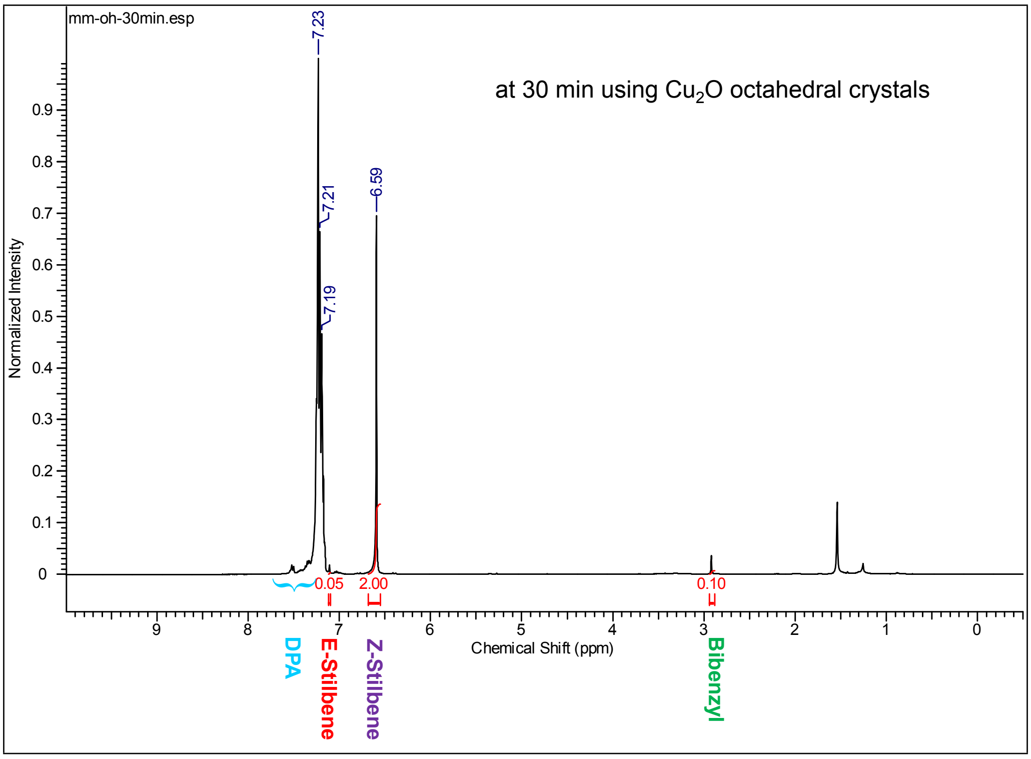
mm-oh-30min.esp
at 30 min using Cu_2O octahedral crystals
7.23
7.21
7.19
6.59
Normalized Intensity
0.9
0.8
0.7
0.6
0.5
0.4
0.3
0.2
0.1
0
0.05
2.00
0.10
9
8
7
6
5
4
3
2
1
0
Chemical Shift (ppm)
DPA
E-Stilbene
Z-Stilbene
Bibenzyl

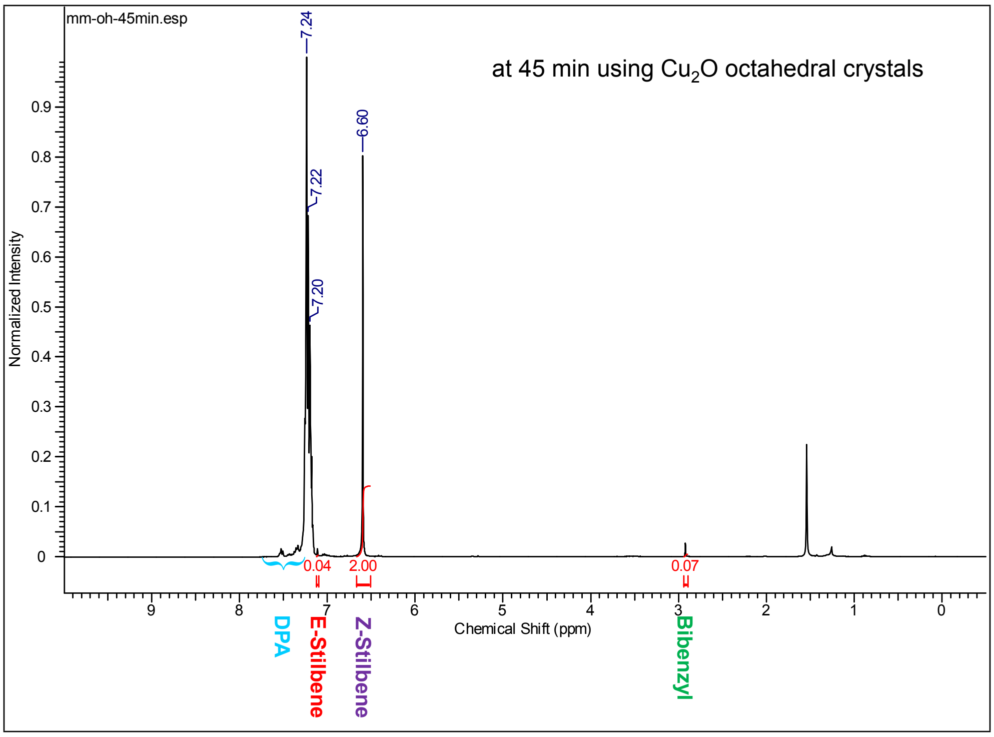
mm-oh-45min.esp
at 45 min using Cu_2O octahedral crystals
7.24
7.22
7.20
6.60
Normalized Intensity
0.9
0.8
0.7
0.6
0.5
0.4
0.3
0.2
0.1
0
0.04
2.00
0.07
9
8
7
6
5
4
3
2
1
0
Chemical Shift (ppm)
DPA
E-Stilbene
Z-Stilbene
Bibenzyl

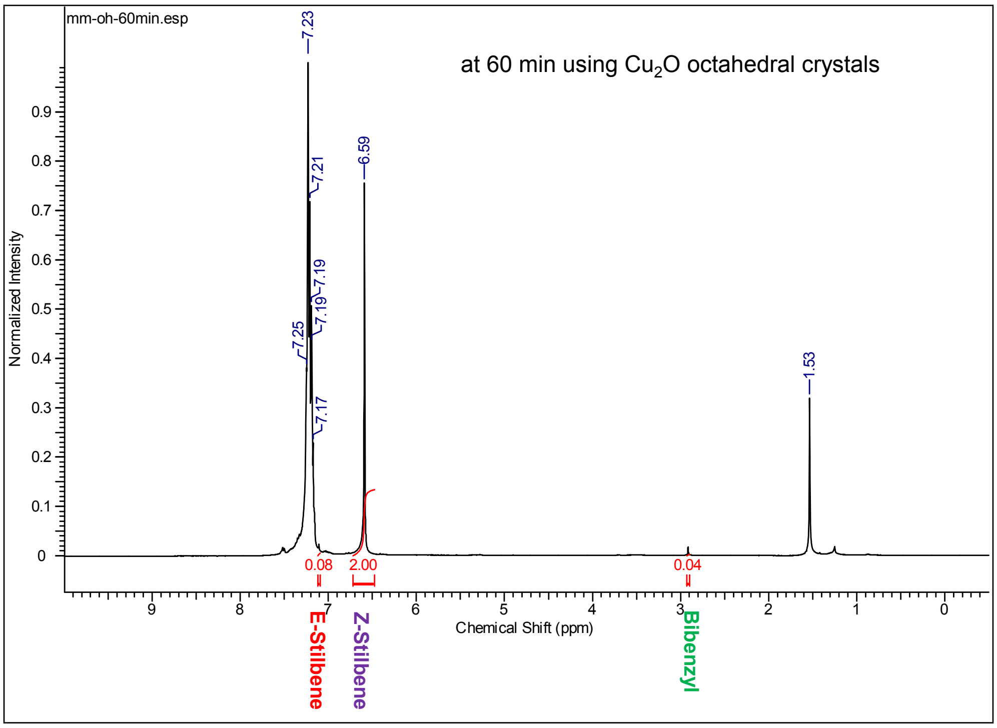

Fig. S17. Product selectivity of Cu octahedra. Time-dependent ^{1}H-NMR study for semihydrogenation of DPA using Cu_2O octahedra. No column chromatography was performed.

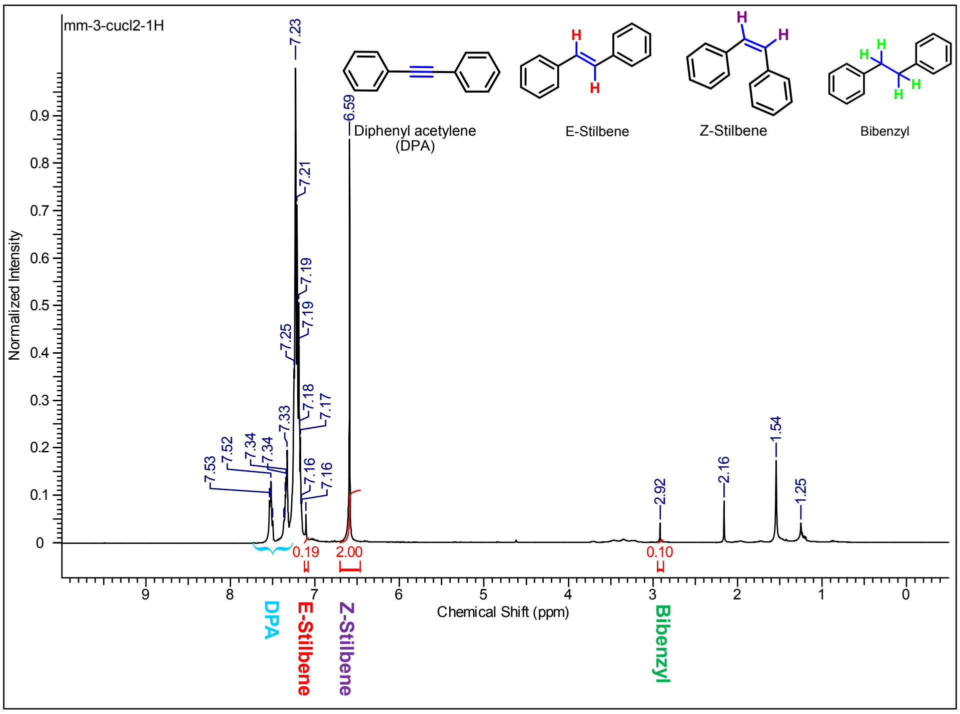

Fig. S18. ^{1}H-NMR spectrum for semihydrogenation of DPA using $CuCl_2$ at 50 ºC for 60 min. $CuCl_2$ was homogeneously dissolved to give Cu ions. Considerable amount of unreacted DPA and formation of (*E*)-stilbene and bibenzyl by $CuCl_2$ shows its inferior product selectivity. No column chromatography was performed.

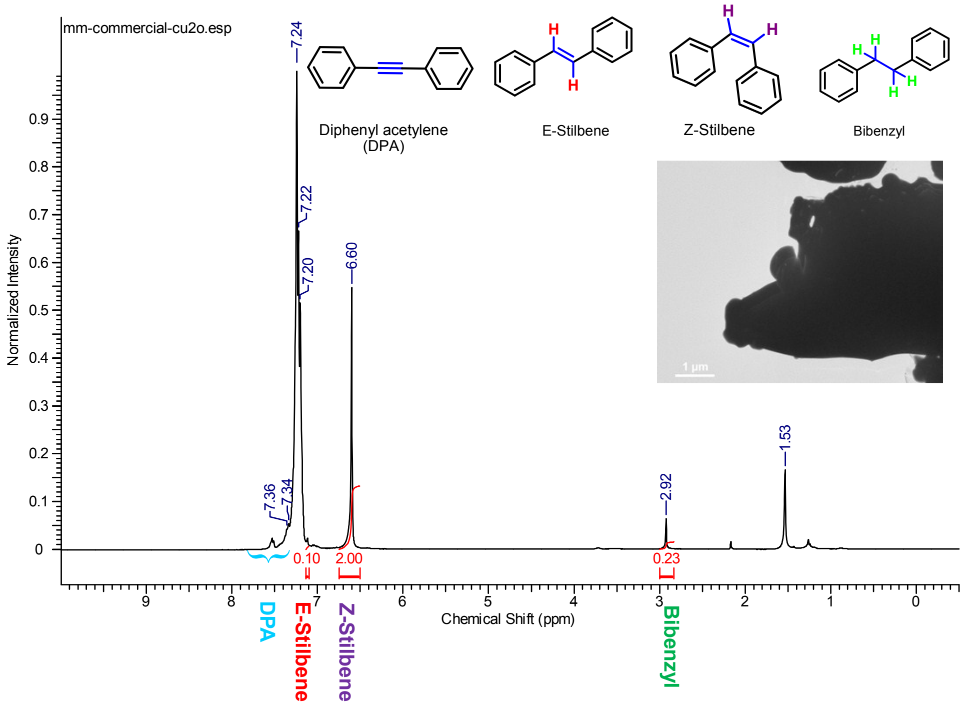

Fig. S19. ^{1}H-NMR spectrum for semihydrogenation of DPA using commercial Cu_2O at 50 °C for 45 min. Commercial Cu_2O particles have no size and shape control. No column chromatography was performed. Inset shows particle TEM image.

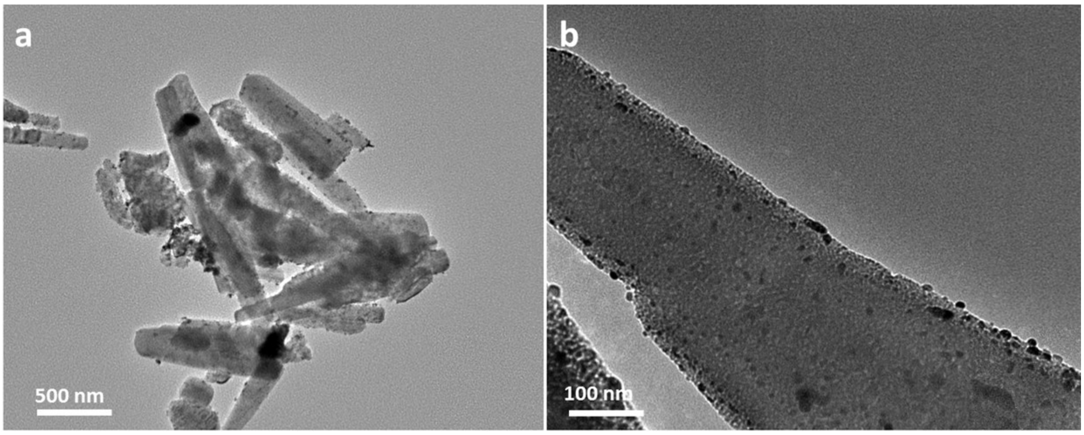

Fig. S20. Lindlar catalyst. (a) Large-area and (b) magnified TEM images of commercial Lindlar catalyst. Pd nanocrystals are the black spots on the $CaCO_3$ support.

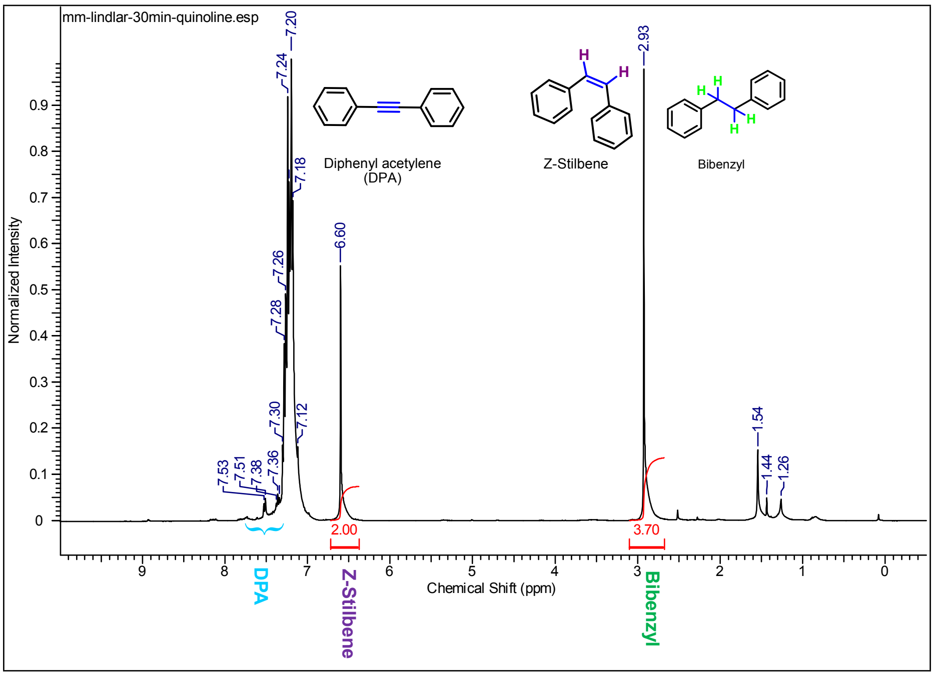

Fig. S21. ^{1}H-NMR spectrum for semihydrogenation of DPA at 50 ºC for 30 min using commercial Lindlar catalyst, taken after the removal of excess quinoline. No column chromatography was performed. The results show unsatisfactory selectivity toward (*Z*)-stilbene formation. Due to the less product selectivity for semihydrogenation of alkynes, further surface modification of Pd-based nanocrystals is usually necessary.

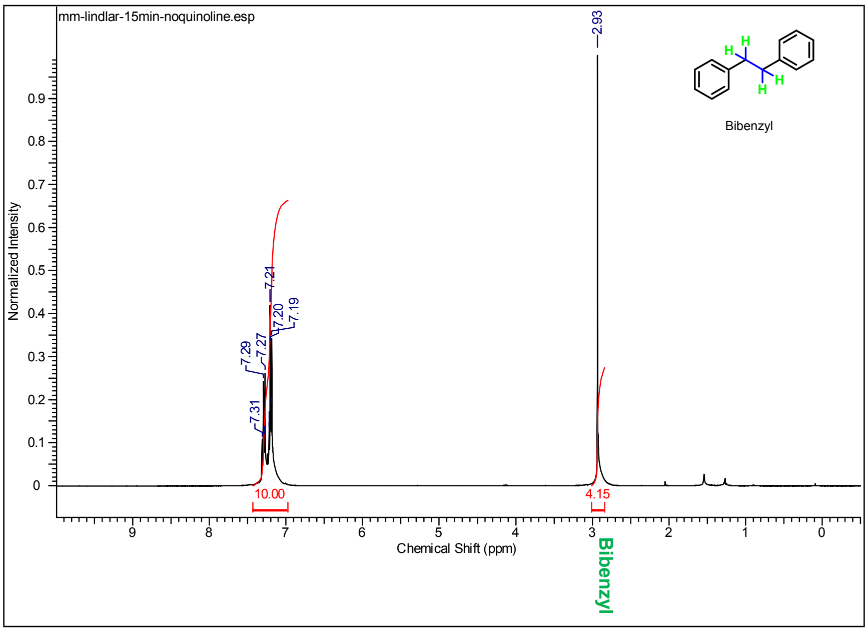

Fig. S22. ^{1}H-NMR spectrum for semihydrogenation of DPA using commercial Lindlar catalyst with AB but no quinoline added, taken after 15 min of reaction at 50 ºC using same catalytic condition for Cu RD crystals. No column chromatography was performed.

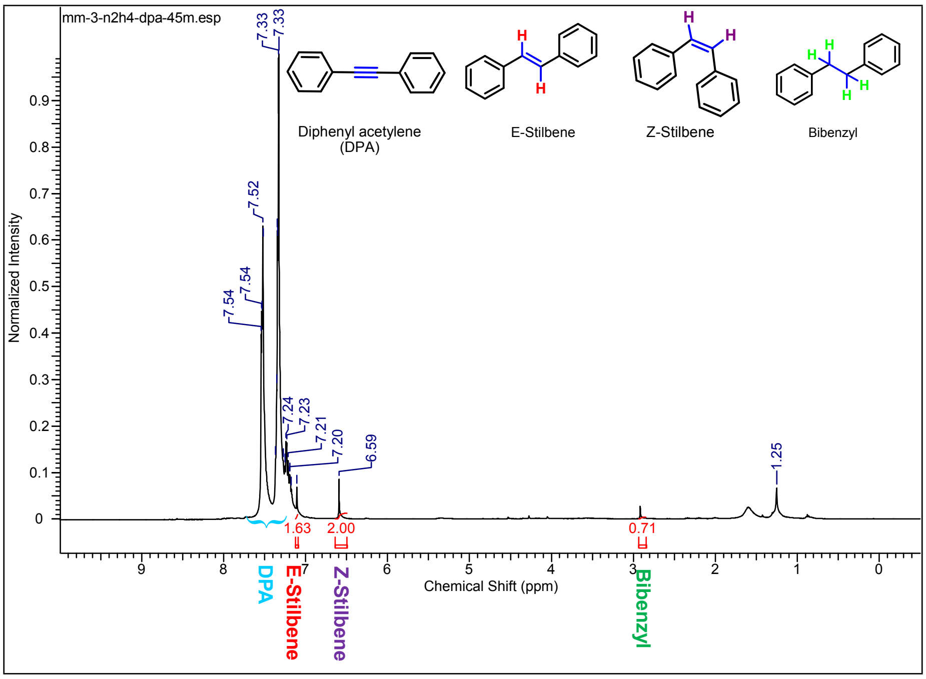

Fig. S23. ^{1}H-NMR spectrum for semihydrogenation of DPA using Cu_2O RD crystals and hydrazine as the reducing agent instead of AB. The spectrum was taken after 45 min of reaction. No column chromatography was performed.

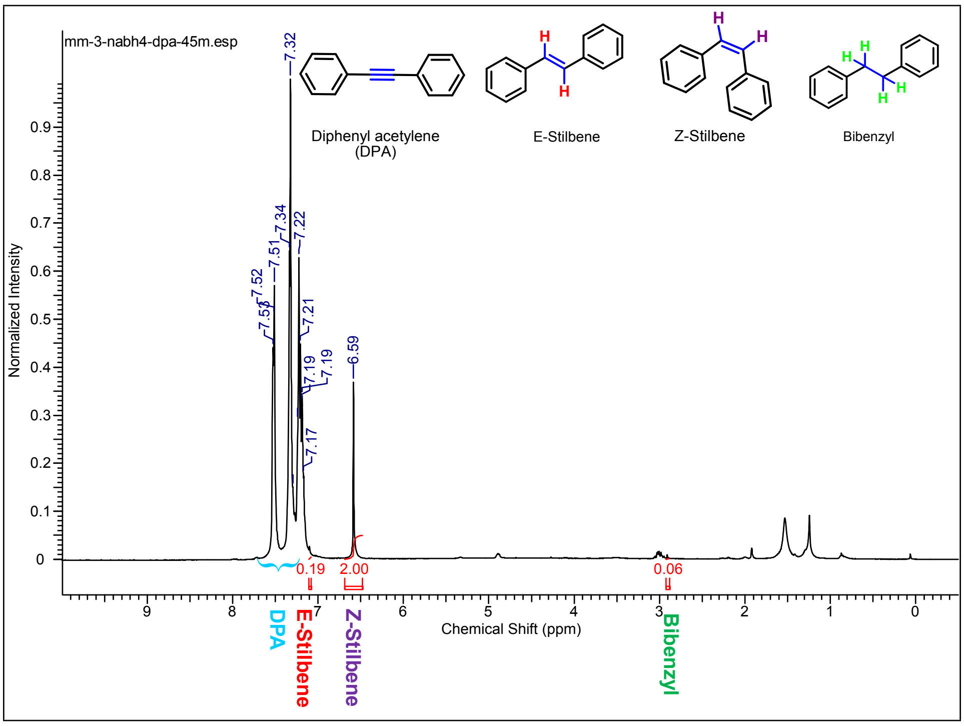

Fig. S24. ^{1}H-NMR spectrum for semihydrogenation of DPA using Cu_2O RD crystals and sodium borohydride as the reducing agent instead of AB. The spectrum was taken after 45 min of reaction. No column chromatography was performed.

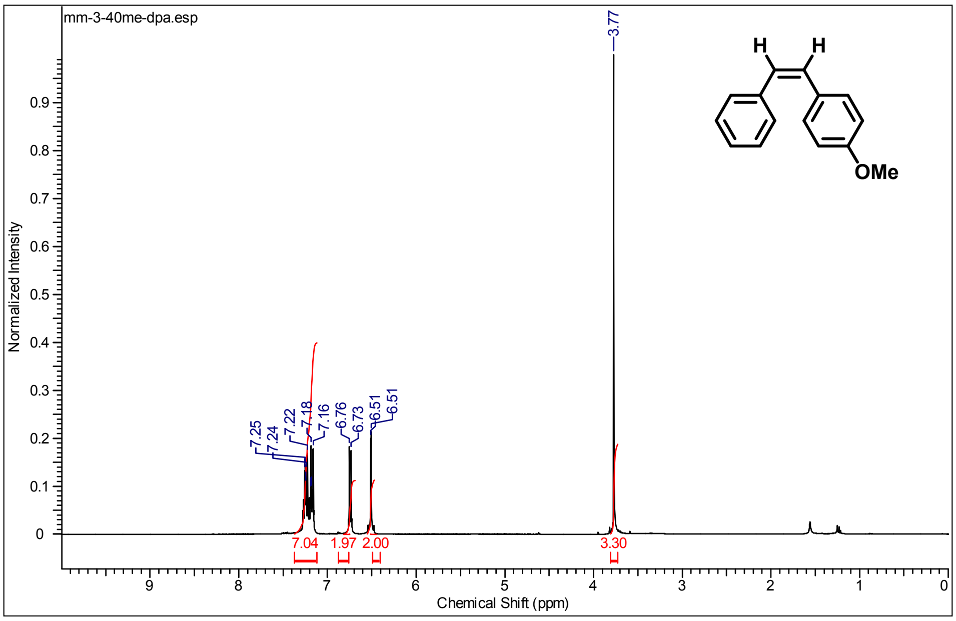

Fig. S25. Other semihydrogenation examples. ^{1}H-NMR spectrum for semihydrogenation of 1-methoxy-4-(phenylethynyl)benzene using Cu_2O RD crystals. No column chromatography was performed.

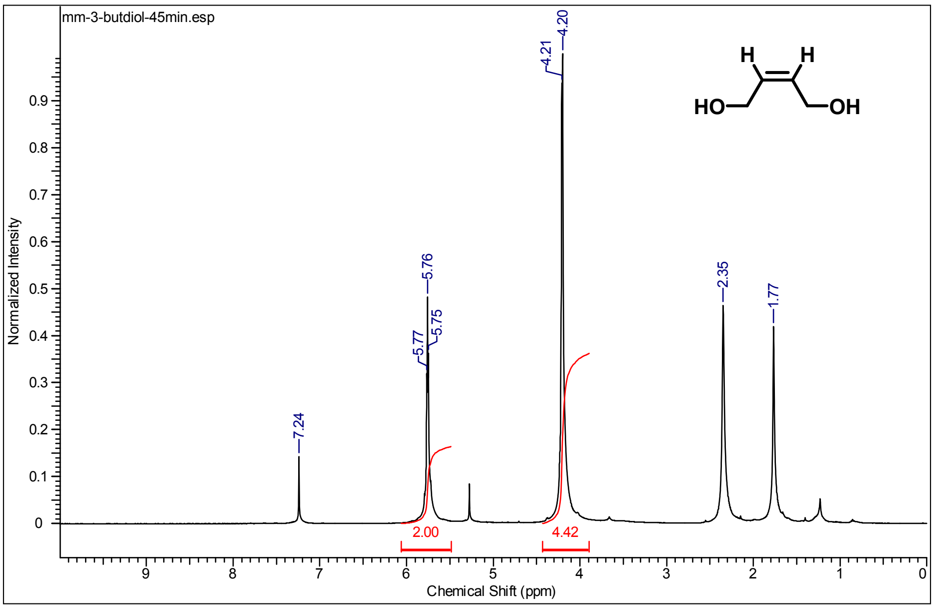

Fig. S26. Other semihydrogenation examples. ^{1}H-NMR spectrum for semihydrogenation of 1,4-butynediol using Cu_2O RD crystals and sodium borohydride as the reducing agent. No column chromatography was performed.

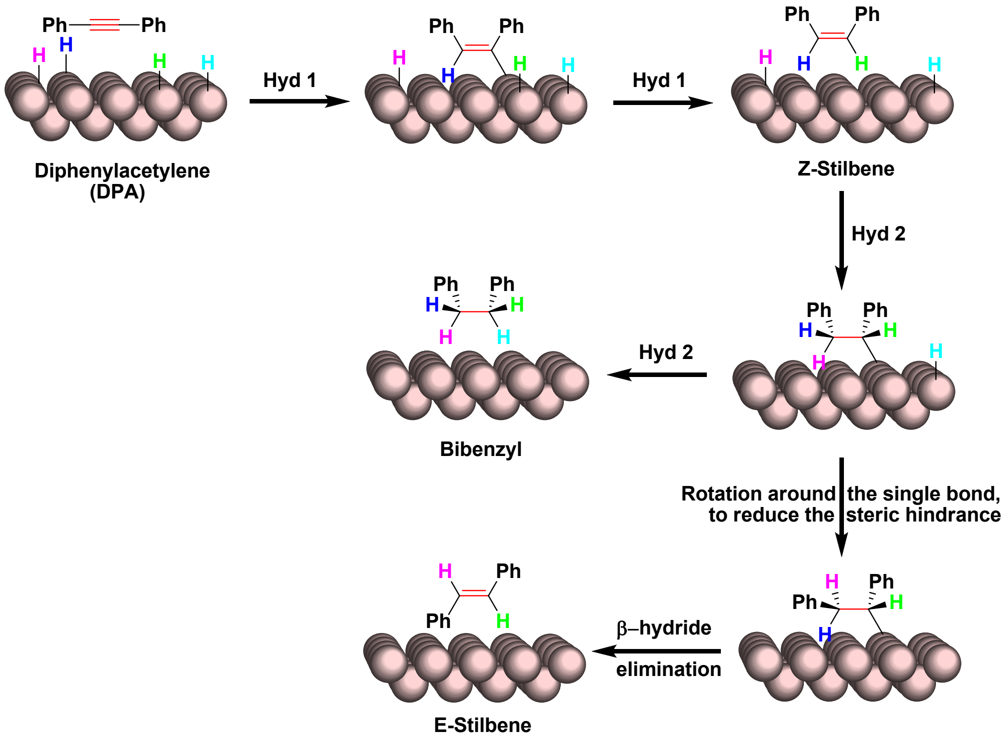

Fig. S27. Mechanistic insight. Representative Horiuti-Polanyi hydrogenation mechanism for the formation of (*Z*)-stilbene, (*E*)-stilbene, and bibenzyl from DPA on Cu (110) surface.

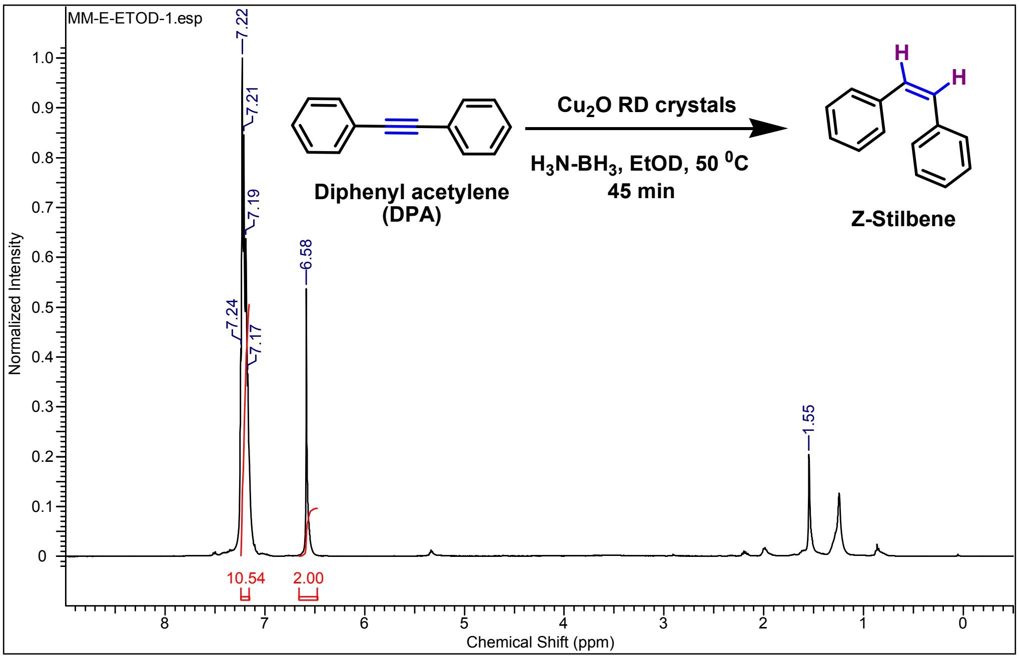

Fig. S28. Deuteration reaction. ^{1}H-NMR spectrum for semihydrogenation of DPA using Cu_2O RD crystals as the catalyst and anhydrous EtOD as the solvent. No column chromatography was performed.

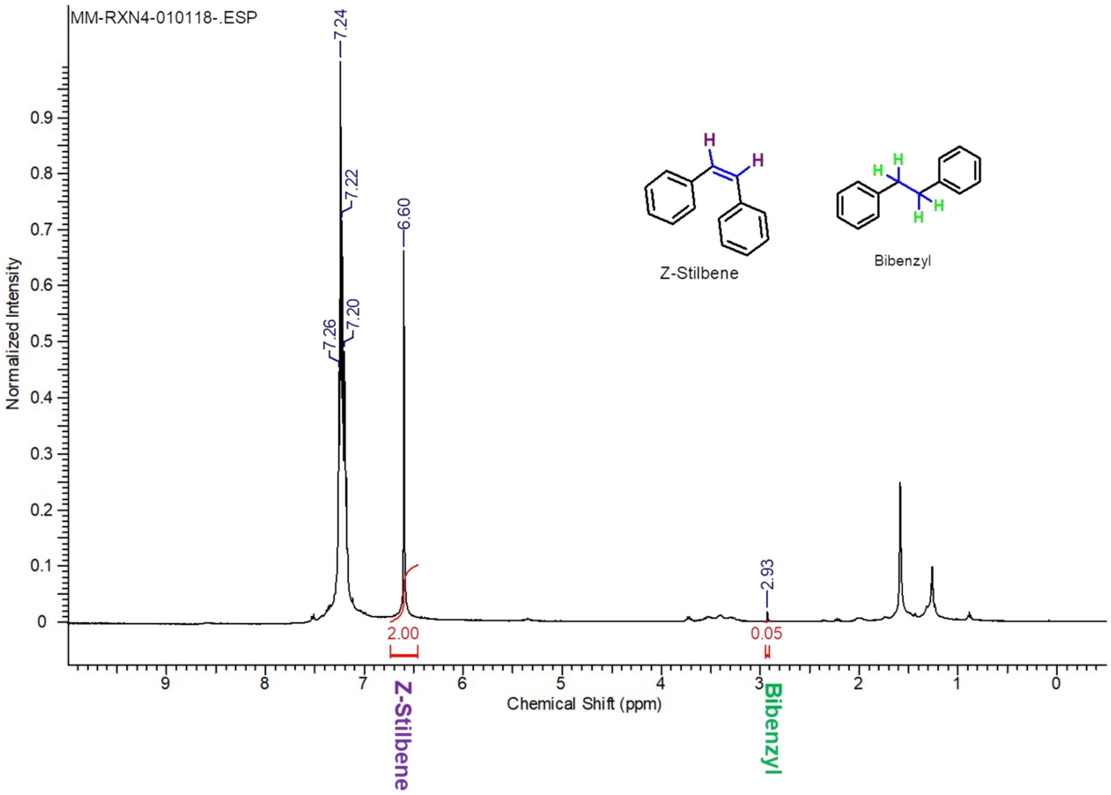

Fig. S29. ^{1}H-NMR spectrum for the hydrogenation of (*Z*)-stilbene under our standard catalytic condition in the presence of Cu RD crystals. No conversion of (*Z*)-stilbene was observed after 45 min of reaction at 50 ºC in the presence of ammonia borane. There is no significant increase in the bibenzyl peak, proving that (*Z*)-stilbene has no strong affinity to bind on the Cu RD crystal surface. We used the (*Z*)-stilbene obtained from our reaction without any further purification. No column chromatography was performed.
